# The Criticality of Consciousness: Excitatory–Inhibitory Balance and Dual Memory Systems in Active Inference

**DOI:** 10.3390/e27080829

**Published:** 2025-08-04

**Authors:** Don M. Tucker, Phan Luu, Karl J. Friston

**Affiliations:** 1 Brain Electrophysiology Laboratory Company, 440 E. Broadway, Suite 200, Eugene, OR 97401, USA; phan.luu@bel.company; 2 Department of Psychology, University of Oregon, Eugene, OR 97403, USA; 3 Department of Imaging Neuroscience, UCL Queen Square Institute of Neurology, London WC1N 3AR, UK; k.friston@ucl.ac.uk; 4 VERSES AI Research Lab, Los Angeles, CA 90016, USA

**Keywords:** excitatory, inhibitory, consciousness, active inference, criticality

## Abstract

The organization of consciousness is described through increasingly rich theoretical models. We review evidence that working memory capacity—essential to generating consciousness in the cerebral cortex—is supported by dual limbic memory systems. These dorsal (Papez) and ventral (Yakovlev) limbic networks provide the basis for mnemonic processing and prediction in the dorsal and ventral divisions of the human neocortex. Empirical evidence suggests that the dorsal limbic division is (i) regulated preferentially by excitatory *feedforward* control, (ii) consolidated by REM sleep, and (iii) controlled in waking by phasic arousal through lemnothalamic projections from the pontine brainstem reticular activating system. The ventral limbic division and striatum, (i) organizes the inhibitory neurophysiology of NREM to (ii) consolidate explicit memory in sleep, (iii) operating in waking cognition under the same inhibitory *feedback* control supported by collothalamic tonic activation from the midbrain. We propose that (i) these dual (excitatory and inhibitory) systems alternate in the stages of sleep, and (ii) in waking they must be balanced—*at criticality*—to optimize the active inference that generates conscious experiences. Optimal Bayesian belief updating rests on balanced feedforward (excitatory predictive) and feedback (inhibitory corrective) control biases that play the role of prior and likelihood (i.e., sensory) precision. Because the excitatory (**E**) phasic arousal and inhibitory (**I**) tonic activation systems that regulate these dual limbic divisions have distinct affective properties, varying levels of elation for phasic arousal (**E**) and anxiety for tonic activation (**I**), the dual control systems regulate sleep and consciousness in ways that are adaptively balanced—around the entropic nadir of **E**–**I** criticality—for optimal self-regulation of consciousness and psychological health. Because they are emotive as well as motive control systems, these dual systems have unique qualities of feeling that may be registered as subjective experience.

## 1. Introduction and Overview

Progress in the understanding of neural dynamics is allowing new integrative theories for how conscious experience might arise in the distributed operations of the cerebral cortex. A key theme is the role of prediction and working memory, such that consciousness serves as an integrator of working memory in ongoing cognition [1]. Considering the neural networks that could support the integrative function of widespread cortical networks, global neuronal workspace (GNW) theory [2] suggests how the synchronization or ignition of coherence within widespread frontal and parietal networks could facilitate this coherent, integrative processing.

In addition to grounding the study of consciousness in the properties of distributed processing in neural networks, other approaches have considered the subjective properties of conscious experience that must be explained by a scientific theory. Reasoning that it is the information itself that must support the properties that are required for consciousness, integrated information theory (IIT) has identified consciousness with the notion of integrated information [3]. Although this “integrated information” is a formal notion, we can see the relevance to the holistic grasp of the meaning of a situation that consciousness must be able to provide.

The theoretical scope and thus distance from specific empirical tests necessary for ITT’s abstract characterization of brain function appears to be challenging for many in neuroscience who are more familiar with objective hypotheses and closely defined experimental tests. A group of neuroscientists and allied academics have recently petitioned to have IIT declared as unfalsifiable and thus unscientific [4,5]. Although this seems to be a somewhat dramatic gesture, one can perhaps understand that falsification may appear particularly difficult to those considering a model like IIT, where what is to be explained are qualities of consciousness defined by subjective experience [6].

### 1.1. Subjective Experience as Evidence for a Theory of Consciousness

The issue in this controversy seems to be whether IIT, accepting as it does the qualities of subjective experience as important evidence of consciousness, can be considered as scientific inquiry. Before behaviorism, the teaching of psychology, such as by William James [7] instructed students to engage in careful reflection on subjective experience. James observed that his own conscious reflections were transient, occupying a period of about 15 s before moving on to a new focus. He described his experience as if it were a bird, moving from these *perchings* of consciousness with *flightings* to new topics. He also used the metaphor of a *stream of consciousness*, and remarked that it often included subjective feelings of personal value, with some ideas experienced as *furtherances*, moving in the right direction, and others as *hindrances*, holding him back.

For many modern neuroscientists and cognitive scientists, and the students they teach, these observations about conscious experience have been relegated to the subjective domain, and thus not allowed as objective scientific evidence. This may be logically problematic, given that the topic to be explained is itself a subjective phenomenon.

### 1.2. The Privileged Subjectivity of Qualia

The difficulty of reconciling subjective and objective forms of evidence arises in a different form for certain philosophers who hold that consciousness is ultimately a subjective phenomenon, a “hard problem,” that cannot be reduced to a mechanistic account of neural mechanisms [8]. In this position, accepted as authoritative by a number of neuroscientists, Chalmers emphasizes that his experience of fundamental qualia of perception, such as the sound of middle C on the piano, are subjective properties that are not in a form that could even in theory be reduced to neural mechanisms.

Furthermore, assuming that science cannot explain subjective experience, Nagel claimed that is vapid scientism to assume that all meaningful facets of life can be explained by science, whether through the principles of physics or those of evolutionary biology [9]. In his famous question, “What is it like to be a bat?” Nagel argued that no amount of objective study can tell us the answer, because the bat’s subjectivity is fundamentally unapproachable by human experience, with no insight possible from scientific inquiry [10].

### 1.3. The Feeling of Consciousness

By these accounts, scientific objectivity and personal subjectivity are irreconcilable. At the same time, some recent scientific theories of consciousness seem to have confused the picture further by proposing that the objective basis for conscious experience may be *found* in subjective feelings. These theories propose a causal status to subjective feelings for generating consciousness. From his extensive study of clinical disorders, Damasio famously described consciousness as *the feeling of what happens* [11]. In his analysis of the levels of experience supported by brain systems, Damasio argues that feeling provides the foundation for experience, perhaps consistent with the classical notion that the core of consciousness is sentience.

Solms drew upon the evidence that subcortical structures, integrated at the level of the midbrain [12], form a sufficient basis for basic consciousness, with the cerebral cortex playing a secondary role [13]. Solms describes both the essential role of the midbrain in regulating alertness and arousal, and he provides compelling case studies of cortical agenesis in children whose social comportment, while limited, manifests many of the features we would assume require intact consciousness.

An equally explicit anatomical analysis—implying that feelings must be the basis for consciousness—has been asserted by Chanes and Barrett, who proposed that consciousness emerges from the global integration of access for neocortical function by the motivationally charged limbic system [14,15].

### 1.4. Computing Experience

Recent information theoretic accounts of cognitive processing have considered Bayesian computation [16,17,18,19] that can be aligned directly with the brain’s distributed processing [20,21]. The description of cognition as *active inference* [22,23,24] has provided a formalism for how the organism’s existing knowledge (i.e., Bayesian priors) provides a (generative) model for effective predictions of environmental states from observable data (sensations) that are assimilated via a neuronally instantiated Bayesian belief updating process.

In a way that aligns well with Helmholtz’s notion of *unconscious inference* in perception, Bastos and associates have detailed how active inference can be related to hierarchical predictive coding in the visual system (drawing on prior perceptual experience, as Helmholtz proposed), with specific roles for both predictions arising from higher cortical areas and error-correction imposed by the constraint of fitting sensory input [25]. Using the same framework—in a way that suggests a novel interpretation of motor control—Adams and colleagues have formulated active inference as the realization of prior intentions, where predictions of the consequences of actions guide lower motor processing through providing predictions [26].

As computational models, these formulations of active inference entail specific and testable theories for their neural implementations, including analysis of the computational basis for consciousness [27]. Furthermore, the very structure of the organism’s information processing, mediating between the intrinsic domain of information that generates affordance predictions and the extrinsic domain that self-corrects in light of environmental data, describes a basis from which consciousness is likely to emerge [28]. With the Bayesian formulation of inference—built on the organism’s self-organization, in relation to sensory evidence—consciousness becomes closely aligned not only with implicit self-evidencing, but explicit self-awareness [29], qualities that are clearly relevant to subjective experience.

At the same time, even a general neurocomputational model of cognition seems a far cry from the notion that consciousness arises from the feeling of what happens. As we will see in this paper, however, as we align active inference with the neurophysiology of corticolimbic mechanisms, it becomes necessary to include the controls on limbic activity from the subcortical arousal and activation systems. These turn out to be well-placed to regulate the feelings of conscious experience, as described by Solms [13].

### 1.5. Regulating Synaptic Connectivity, and Consciousness, with Our Feelings

In this paper, we attempt to extend the accounts of Damasio, Solms, and Chanes and Barrett by arguing that consciousness emerges in the process of active inference through adaptive controls (feelings). We argue that these feelings are emergent from the brain’s arousal and activation systems, based in the limbic and subcortical networks that regulate memory and inference (prediction and error-correction) in the cerebral cortex [30,31,32]. In fact, the dual limbic memory systems have motivational features of feedforward and feedback control that may explain the specific control of prediction and correction, respectively.

The neurocomputational mechanisms of active inference are fundamentally implicit, consistent with Helmholtz’s original insight. In fact, we will see the fundamental inhibitory nature of error correction and the excitatory nature of generating predictions as we examine the consolidation of the dual forms of memory in NREM and REM sleep, respectively. So, how could such implicit, unconscious processes give rise to feelings?

Building on the inhibitory and excitatory neurophysiological mechanisms of memory consolidation in sleep stages [33], we will propose the *criticality hypothesis of consciousness*. This hypothesis suggests that the opponent complementarity of the excitatory (REM-like) and the inhibitory (NREM-like) systems generate consciousness when simultaneously active in waking and balanced at excitatory–inhibitory criticality. To support this proposal, we will outline a control theoretic formulation for regulating conscious processing, where the primary controls are affective biases on the predictive priors and the corrective feedback of active inference (the excitatory and inhibitory feelings of elation and anxiety). Technically, these variables play the role of gain control that can be read as an encoding of confidence or precision; equipping active inference with ‘mental actions’ and a putative phenomenology [34,35,36,37,38,39,40].

We will argue that the mechanisms of active inference can then explain key qualities of consciousness: the subjective sense of what happens next (excitatory prediction) balanced against the objective experience of what just happened (inhibitory correction). These dual aspects of active inference are each motivated by ‘feelings.’ Indeed, the experience of neural excitation is predictable: we experience it as degrees of elation, a cardinal dimension of subjective affect [41]. There is psychological evidence that the excitation of prediction and thus the experience of the future are engaged by at least moderate degrees of elation [42]. Similarly, the sensitivity to error-correction and self-restraint are associated with the second cardinal dimension of emotion; namely, anxiety and/or hostility [41]. The inhibition integral to the neural mediation of anxiety is a second (i.e., complementary) affective control system essential for sense-making under uncertainty [42].

Thus, the phasic arousal (depression to elation) and tonic activation (security to anxiety) systems can be recognized in subjective experience by their emotional qualities, their valenced affects. We hypothesize that these excitatory and inhibitory feelings create the ‘grip’ on information we experience as consciousness, when they are balanced.

However, a constant state of balance is not adaptive; we need to be more elated sometimes, to be flexibly creative, and more anxious at other times, to be cautiously critical. Adaptive awareness then allows contextual active inference when the excitatory and inhibitory feelings are engaged in varying degrees. The result of the feeling of what happens [11] is then a subjective explanation for experience [43]. The requisite variations are affectively charged periods of relative excitation or inhibition, generating feelings (phasic arousal of elation and/or tonic activation of anxiety) that then align directly with William James’s experiences of furtherance and hindrances [7]. Crucially, for adaptive variations around these opponent processes, they need to be balanced in a critical regime. This is the basis of the criticality hypothesis pursued below.

### 1.6. Unconscious Self-Organization in Sleep

In addition to the optimization of (Bayesian) belief updating by variations in excitatory (predictive and constructive) and inhibitory (revisionary and critical) processing during waking, the day’s lessons and opportunities for active inference continue through major excursions from criticality in sleep [33]. Here the excitatory and inhibitory operations of ‘affective inference’ are manifest in their fundamentally distinct forms. We describe this as affective inference because the controls are motivational systems for regulating active inference in (subsequent) perception and behavior.

In waking, we make predictions about what’s happening, and then we correct these predictions on the basis of what happened [44]. In sleep, first we correct our earlier predictions with the inhibitory control of NREM sleep. The salient unpredicted events of the day seem to be held in a kind of limbic resonance, and—with the onset of NREM sleep—these events are replayed for refining the generations of predictions through the inhibitory neurophysiology of Non-REM (NREM) sleep. Following each NREM episode, Rapid Eye Movement (REM) sleep then breaks criticality, through excessive excitement that optimizes and reconsolidates the organism’s Bayesian priors to optimize the generative capacity (i.e., generalization) for prediction in the next day’s active inference [33].

These dual organizational mechanisms of sleep—that recapitulate neurodevelopmental self-organization [45]—are then linked to waking experience by controls engaged by the ongoing excitatory and inhibitory processes of active inference in conscious waking. In this way, the alternating nighttime excursions from criticality organize the brain’s generative model (with inhibitory feedback during NREM and excitatory feedforward exercise in REM) to restore synaptic homeostasis, and ensure criticality to support the next day’s waking consciousness [46].

### 1.7. Affective Control of Criticality

Considering both the minor excitatory–inhibitory (**E**–**I**) excursions of adaptive waking, and the major excursions of sleeping, the criticality hypothesis of consciousness proposes that active inference can explain the essential feelings of subjective experience. We self-organize to a critical regime of belief updating through the **E**–**I** balance that underwrites our feelings. Consciousness ignites as an enduring memory (extended current awareness) when the global neuronal workspace can be accessed at criticality.

After reviewing the key empirical evidence and rehearsing the principles of variational inference and accompanying criticality, we conclude by considering experimental predictions, phenomenological implications, and the properties of neurophysiological excitement and inhibition that we recognize as conscious experience. In the next section, we summarize key terms.

## 2. Key Terms

The primary concepts for this theoretical approach are described in Table 1. 

## 3. Adaptive Control from the Dorsal and Ventral Limbic Divisions

The underlying mechanisms of consciousness must include working memory. The role of working memory within current theories of consciousness can be aligned with philosophical accounts of the phenomenology of conscious experience [47]. Working memory involves both the narrow focus of explicit representations that are readily manipulated, as well as the more implicit “activated long-term memory” representations that may be primed for access, if not immediately available in consciousness [48].

William James emphasized that—in addition to the narrow focus of consciousness—there is a wider fringe of consciousness that is an integral resource for ongoing thought [7]. John Dewey proposed that focal consciousness has not only a fringe of not fully aware experience, but also a *field* of unconscious but vaguely activated (intuitive) meaning that informs present experience [49]. These classical phenomenological accounts are more pragmatic than the modern portrayals of subjectively pristine (and therefore mechanistically irreducible) qualia of conscious experience [8,10] that restrict consciousness to the most iconic and singular representations of focal, explicit representation. In contrast, a more pragmatic account may allow a wider range of fringe and field meaning to be allowed into the penumbra of personal consciousness, where the active, dynamic, and motivated mechanisms of working memory may be more intuitively appreciated [50]. Understanding the neural mechanisms of implicit and explicit memory that give rise to these varying states of consciousness may allow a scientific account of the role of consciousness in a full range of *adaptive* behavior, as implemented through motivational and emotional biases that direct active inference toward goals or away from threats.

### 3.1. Dual Memory Systems

In considering the dynamic range of activation of associations within working memory that contribute to consciousness, modern research can draw on computational models of distributed representations in which information is represented through the strength of synaptic connections within cerebral networks. Knowing the network architecture of the mammalian cortex, such as formulated within the Structural Model [51], enables explicit analysis of how this connectional architecture must support parallel distributed processes, including differing forms of access to working memory.

**Figure 1:**
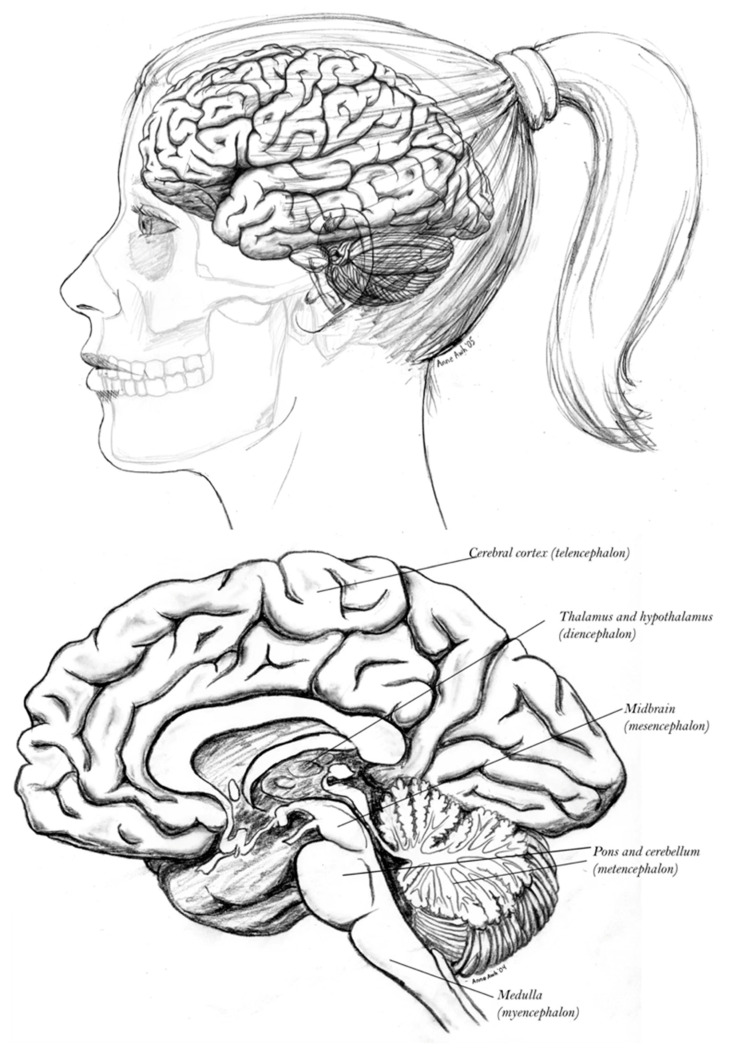
The human brain includes the left (**top**) and right (**bottom**) cerebral hemispheres, including large wrinkled sheets of cortex. The base of the brain is composed of the brainstem rhombencephalic (myencephalon and metencephelon) and midbrain mesencephalic arousal control systems. At the inner core of each cerebral hemisphere is the limbic system, interfacing the hemisphere with the subcortical levels that provide both motivation and arousal controls for the entire brain.

Situated at the core of the cerebral hemisphere (Figure 1), the limbic system is densely interconnected both with subcortical control systems of the hypothalamus and thalamus (connecting to midbrain and brainstem) as well as the extensive heteromodal cortex adjacent to the limbic networks, which are interconnected with not only frontal and temporoparietal networks, but also unimodal association cortices and primary sensory and motor areas as detailed in the Structural Model. As a result, corticolimbic interactions (and their thalamic mediation) are central to both ongoing working memory and the consolidation of enduring long-term memory within the distributed connectivity of the human cortex [52].

**Figure 2:**
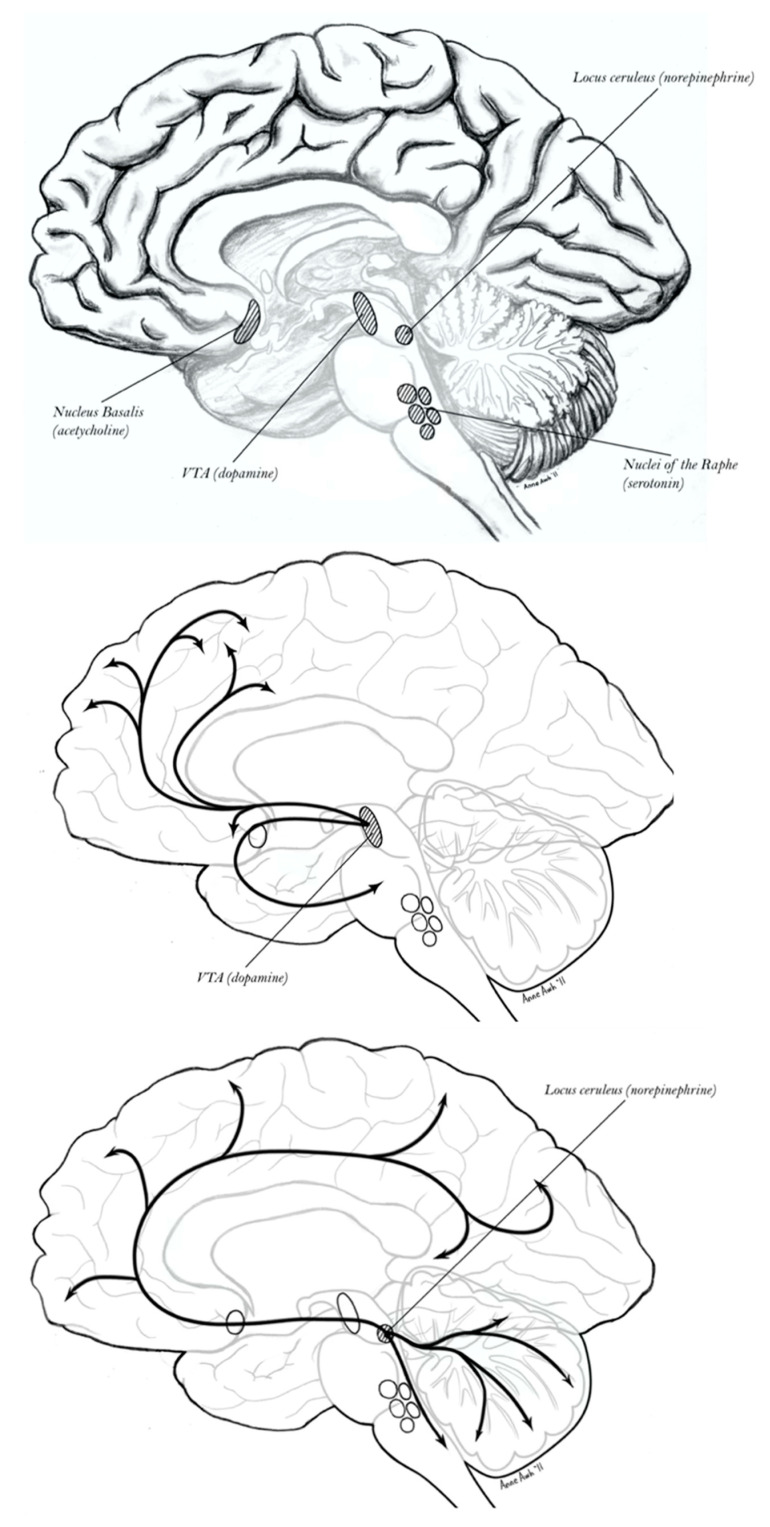
The regulation of sleep and consciousness in the human brain is controlled by major neuromodulator systems located in the brainstem (serotonin and norepinephrine, midbrain (dopamine) and basal forebrain (Nucleus Basalis acetylcholine). These are, in turn, regulated by the wake-sleep state switching and circadian regulators in the hypothalamus, including the orexin/MCH balance for regulating the daily wake-REM cycle and adenosine for regulating NREM sleep pressure.

The neural activity and cognitive operations of the cerebral hemispheres, in sleeping and waking, are regulated by the neuromodulator-specific brainstem and midbrain arousal control systems, together with the forebrain nucleus basalis (Figure 2). These are in turn regulated in complex patterns by the telencephalic hierarchy of cortical, limbic, striatal, and thalamic systems.

Both traditional neurology and modern cognitive neuroscience have largely treated the mnemonic functions of the limbic system as separate from motivational functions, even though it is clear in the animal literature that mammalian memory is adaptively motivated [53,54]. In the clinical syndromes of cortical lesions, both emotional and mnemonic functions are typically damaged by limbic lesions. There is a clear picture of the dual memory systems from amnesias that are common not only with dorsal limbic (particularly the hippocampal) lesions, but also ventral limbic (insula and amygdala) damage [55,56].

**Figure 3:**
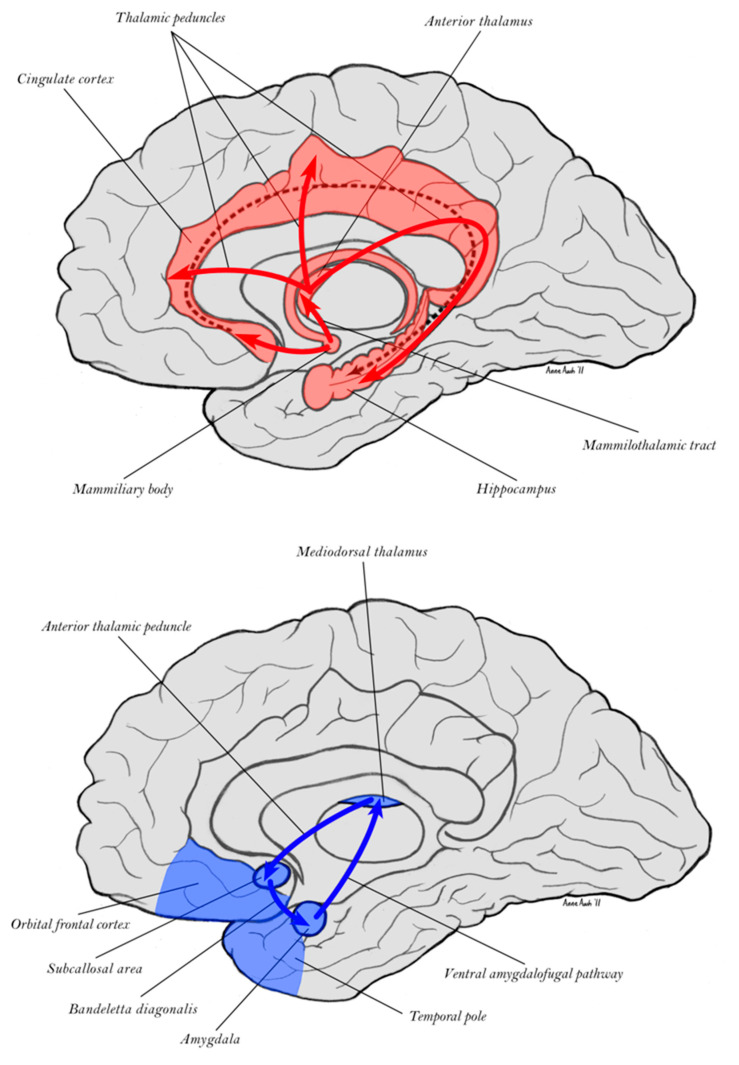
Dorsal and ventral divisions of the limbic system. **Top**: Dorsal (Papez) limbic circuit linking hippocampus with cingulate cortex, septum, and mammillary body of the hypothalamus, with regulation by the anterior nucleus of the thalamus. This anterior thalamic nucleus receives lemnothalamic projections from the pontine brainstem. **Bottom**: Components of the ventral (Yakovlev) limbic circuit linking the amygdala, anterior temporal pole, and caudal orbital frontal cortex through the mediodorsal nucleus of the thalamus. The mediodorsal thalamic nucleus receives collothalamic projections from the midbrain.

The dorsal limbic system is comprised by the Papez circuit (colored red in Figure 3), regulated by the mammillary body of the hypothalamus and the anterior nucleus of the thalamus, and engaging the hippocampus, cingulate cortex, and septum [54,57]. Complementing this division, the ventral limbic Yakovlev circuit includes the amygdala, anterior temporal pole, and orbital frontal cortex, regulated by the mediodorsal nucleus of the thalamus, including the unique (one-directional) triangular circuit shown in blue in Figure 3 [58,59,60].

Closely aligned with the limbic memory functions are the motivational and emotional biases that are manifested in pathological forms, depending on whether dorsal or ventral divisions are damaged. Dorsal limbic lesions, such as to cingulate cortex, often result in *pseudodepression*, involving a loss of optimistic motive engagement and hope [42,61]. Ventral limbic network damage, on the other hand, such as with lesions to anterior temporal and orbital frontal networks, typically leads to a *pseudospsychopathic* syndrome, in which inadequate anxiety leads to poor impulse control and social insensitivity [42,61]. The classic animal model of ventral limbic damage is the Kluver-Bucy syndrome [62], in which removal of the anterior temporal lobe, damaging the extended amygdala [63] in monkeys, leads to hyper-oral and hyper-sexual behavior (releasing the hedonic impetus of the dorsal limbic division). It also produces what Kluver and Bucy called *hypermetamorphosis*, a sensitivity to each change in the environment, reflecting the exaggerated, feedforward orienting to novelty caused by the dorsal limbic release.

Studying these effects—together with congruent effects of limbic lesions or stimulation in animal learning [64], as well as the unique dorsal and ventral frontolimbic contributions to control of motor behavior [65]—led Tucker & Luu [42] to propose that dorsal limbic motive control is associated with the phasic arousal of the excitatory affect of elation, leading to an *impulsive* optimism such as is exaggerated in clinical mania. This can be considered as the excitatory *impetus* for actualizing organismic affordances. In normal brain function, this bias is balanced by the ventral limbic mode of *constraint* and inhibitory anxiety regulated by the tonic activation system, described as an inhibitory constraint, the *artus*, which in humans enhances sensitivity to social feedback for developing self-control of personal behavior [42].

### 3.2. Active Inference in the Cerebral Cortex

Bringing these differential limbic motive biases to the evolved architecture of action regulation in the cerebral cortex, Tucker & Luu [31] proposed that the emotionally positive, feedforward bias of the dorsal limbic division and the associated dorsal neocortex may be important to generating predictions and expectancies in the limbifugal (from limbic) direction in the process of active inference [25]. The dorsal division of neocortex has a minimally developed granular layer [66], suggesting it is less constrained by inhibitory control than the ventral neocortex, which has a well-developed granular layer 4 with inhibitory interneurons that are important for error-correction in the limbipetal (toward limbic) direction of active inference [25].

**Figure 4:**
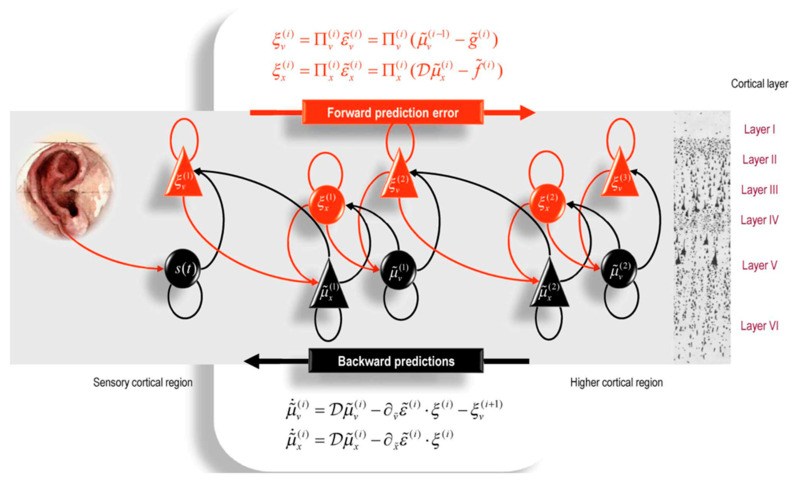
From Friston, Breakspear, & Deco, [23]: Schematic detailing a neuronal architecture that might encode conditional expectations about the states of a hierarchical model. This shows the speculative cells of origin of forward driving connections that convey prediction errors from a lower area to a higher area and the backward connections that construct predictions (Mumford, [16,17]). These predictions try to explain away prediction errors at lower levels. In this scheme, the sources of forward and backward connections are superficial and deep pyramidal cells, respectively. The equations represent a generalized descent on free energy under the hierarchical model described in the main text. State-units are in black and error-units in red. Here, neuronal populations are deployed hierarchically within three cortical areas (or macro-columns). Within each area, the cells are shown in relation to cortical layers: supra-granular (I–III) granular (IV) and infra-granular (V–VI) layers, as shown in the illustration of a eulaminar section of cortex at right.

Figure 4 illustrates how active inference might work in cortical hierarchies in auditory perception, organizing communication across higher cortical areas (such as heteromodal cortex) at right, unimodal association cortex (in the middle) and primary auditory cortex at left [23]. The feedforward expectations for what should be heard (*predictions* in black) are passed from higher to lower areas (black state units and black arrows), where the feedback corrections (prediction *error* in red) from the sensory data are processed to update the predictions on the basis of sensory errors. Note that this illustration in Figure 4 uses the conventional neuroscience assumption of “forward” information flow starting with sensory input, whereas in control theory terms [52] this sensory updating is better described as *feedback* control, which is the usage we maintain throughout this paper.

Interpreting active inference as a motivated process, regulated by the limbic base of the neocortical hierarchy, we can see how the generation of predictions in the limbifugal direction is motivated by the organismic feedforward bias of motive excitement (degrees of subjective elation) in the dorsal limbic division [31]. This control by Bayesian priors is complemented by the error-correction in the limbipetal direction, regulated uniquely by the feedback constraint bias of inhibition (subjectively experienced as degrees of anxiety) applied by the ventral limbic division [31].

### 3.3. Dorsal Excitatory and Ventral Inhibitory Controls on Attention and Working Memory

In the literature on the functional connectivity of cortical networks [67,68], the dorsal and ventral attention systems provide opponent biases, reflecting internal or endogenous control from the dorsal attention network, in contrast to external or exogenous control for the ventral attention network [69]. Recognizing the limbic control of these major neocortical networks, there is a close correspondence with the formulation of active inference regulated by feedforward and feedback biases [30,31] with the generative process of predictions—based on Bayesian priors—balanced by the corrective regulation of error-correction during evidence accumulation [70,71]. In this setting, the concept of a Markov blanket describes the information surface forming the interface between the intrinsic domain (generating predictions) and the extrinsic domain (mediating sensory feedback from the world).

**Figure 5:**
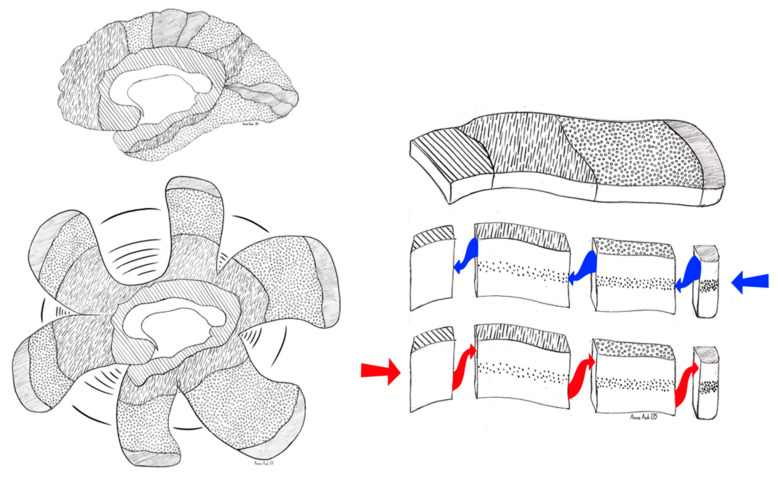
**Left:** Cartoon suggesting how the major sensory and motor pathways of the cortex may be unfolded (here, 3 dorsal and 3 ventral pathways are shown). Striped = limbic; dashed = heteromodal association cortex; stippled = unimodal association; shaded = primary sensory or motor. Lines between pathways reflect the density of cross-pathway connections. **Right: Top:** a single pathway. **Middle:** the input (limbipetal feedback) projections (in blue) in the Structural Model are from supragranular to the higher area granular layer. **Bottom:** the output (limbifugal feedforward) projections (in red) proceed from infragranular (5–6) to supragranular (2–3) layers.

Figure 5 provides a schematic to illustrate how limbic control might be integrated with Bayesian belief updating. Several sensory and motor pathways of the human brain are unfolded at left, illustrating that the integration of cognition—through interconnected heteromodal cortex (dashed)—is regulated adaptively by the limbic base (striped) that represents the still “higher” area of the cortical hierarchy. At right is the predictive processing across the hierarchy of one pathway, reflecting limbifugal (from limbic) flow from infragranular to supragranular layers in the Structural Model (red), indicating the excitatory feedforward control proposed by Tucker & Luu [31,42]. The balancing feedback control from supragranular layers to layer 4—mediating error correction [25]—is shown by blue arrows, reflecting the constraint on perception imposed by sensory data, a feedback process that we propose is regulated by the inhibitory motive control of anxiety [31].

A Markov blanket is then the contact surface between the intrinsic core self and the extrinsic networks of the cerebral cortex mediating exchange with the world. The predictive affordances of the intrinsic brain are served up by the pyramidal projections from 5–6 to the more supragranular layers of the lower (closer to sensory or motor) regions of cortex, where the extrinsic sensory evidence into layer 4 provides corrective constraints to balance the contributions of sensory evidence and prior constraints.

Although we can envisage one Markov blanket—in the sense of an intrinsic-extrinsic boundary—anatomically we can see that there are many facets of the interface, formed between each of the major levels of corticolimbic organization: limbic, heteromodal, unimodal, and primary, where each level is synchronized with the same level of other pathways through lateral connections as shown in Figure 5 at left [72]. Each Markov blanket demarcates the levels of experience and memory in the control of the visceral, cognitive, and sensory-motor process of active inference. Within this architecture, one can appreciate the specific qualities of excitatory limbifugal and inhibitory limbipetal control, negotiating—at each level of the corticolimbic hierarchy—the excitatory realizations of internal affordances, on one hand, with the inhibitory feedback constraints of ongoing contact with the environment, on the other.

### 3.4. Formulating Excitatory Phasic Arousal and Inhibitory Tonic Activation in the Vertical Integration of Working Memory

In this theoretical framework, we are using concepts of excitation and inhibition at a higher level of abstraction than the depolarizing effects of glutamatergic pyramidal neurons (excitatory) and GABAergic interneurons (inhibitory). In other words, there are two kinds of excitation–inhibition balance. The first kind refers to the updating of neuronal representations through error correction, where (ascending, limbipetal) precision-weighted prediction errors cancel themselves by updating higher-order representations that generate (descending limbic) predictions. This kind of excitation–inhibition balance mediates the *first-order* inference about the *content* of representations. However, we are concerned with the *second-order* inference about the *context* in which the first-order representations are updated. This context corresponds to the precision or confidence afforded prior beliefs, relative to sensory evidence. It is in this sense that the (motive) control of precision has to be balanced; otherwise, sensory evidence would overwhelm prior predictions; or vice versa.

The question is whether the properties of **E**–**I** criticality apply at this contextual, second-order level of self-regulating cognition (c.f., metacognition) in the brain. If so, (meta) cognition could be defined in terms of criticality. The theoretical basis of this view can be seen in the distinct motive (i.e., precision) controls that are proposed to underly the mnemonic capacities of the dorsal (Papez) division and ventral (Yakovlev) division of the limbic system. The proposal is that the dorsal division operates under a feedforward, impulsive form of control, thereby exerting an *excitatory* influence on prior predictions [30,31,42]. In tracing the dorsal limbic circuits, including the regulation provided by the anterior nucleus of the thalamus, there are important bidirectional modulatory influences—between the dorsal limbic division and the lemnothalamic pathways—on the phasic arousal mediated by the pontine brainstem [30]. Particularly important to this excitatory, motive-memory system is locus coeruleus norepinephrine (LC NE) modulation (through the dorsal NE bundle) that applies a habituation bias as an integral component of phasic arousal [73]. Integrating this NE habituation bias with the widespread excitatory tuning of the forebrain nucleus basalis cholinergic (NB ACh) projection system [74], the functioning of the dorsal limbic division—and the associated dorsal neocortex—is *excited*, reflecting increased activity in the limbifugal pyramidal pathway generating predictions [31].

Importantly, the NE habituation bias keeps excitation from running out of control. The net effect is then an impulsive form of control; rapidly generated yet quickly habituated. See [42] for empirical evidence on functioning of the dorsal corticolimbic networks.

In a similar but opposite fashion, the inhibitory process of the ventral corticolimbic division is not limited to GABAergic inhibition (which is indeed a key element of the expanded granular layer in ventral neocortex). Rather, it is a modulatory, inhibitory function applied to limbic, striatal, and cortical activity that is *sustained.* It is sustained by dopaminergic (D4, D2) control from the ventral tegmental area of the midbrain, mediated through collothalamic pathways, targeting the ventral limbic, striatal, and cortical areas in particular. The DA regulation, described as tonic activation and also engaging NB ACh, leads to a sustained and active form of inhibitory control, causing representations to be focused and restricted (c.f., representational sharpening) even when input is maintained at a high level.

With this specific form of modulation, greater inhibition does not just shut down neural activity; rather it sustains it in time [73]. The net effect is what Pribram and McGuinness [75] described as the *redundancy bias* of tonic activation. There are, therefore, combined effects of reduced prior precision, and enhanced sensory precision and persistence, that we describe as the cognitive effects of anxiety.

Thus, the regulation of neural activity by phasic arousal and tonic activation can be read as excitatory and inhibitory forms of control, such that their balance—at criticality—may be an essential property for optimal neurophysiological and psychological function.

Even though we have emphasized differences in dorsal and ventral biases, it is important to recognize that there are descending, predictive (excitatory) influences and ascending error-correcting (inhibitory) influences in both dorsal and ventral cortical divisions, and there are important interactions between dorsal and ventral divisions both at the limbic level [76] and the neocortical level [77]. As a result, our emphasis on the excitatory (impulsive) control of the dorsal corticolimbic division, and the inhibitory (constrained) control of the ventral division, is a relative one. Nonetheless, understanding even this relative emphasis on excitatory feedforward versus inhibitory feedback control in the complex human brain may be simplified through appreciating the evolution of this control architecture from earlier vertebrate forms, where excitation and inhibition were managed by more elementary, and interpretable, pallial and subpallial neural architectures and their respective brainstem and midbrain control systems.

### 3.5. Evolution of Mammalian Self-Regulation Through Weaving Excitatory and Inhibitory Architectures

Looking to the subcortical controls for the arousal regulation of the limbic divisions and associated neocortex in mammals, Luu, Tucker, & Friston [33] traced the subcortical pathways and found that the midbrain tonic activation system and its dopaminergic neuromodulator substrate are particularly important to the ventral limbic division, providing the source of inhibitory constraint—associated with anxiety—for the ventral network. In earlier ancestors (amphibians and reptiles), the DA-regulated midbrain-collothalamic pathway targeted the subpallium (basal ganglia) exclusively. In mammals this collothalamic targeting includes cortical-pallial as well as subpallial territories. Yet in mammals, we still see the collothalamic foundations in the inhibitory mechanisms of the striatum operating on the limbic system and cortex. Whereas the matrix division of the striatum targets the cortex, the patch (striosome) division has its major projections to the midbrain DA control centers (substantia nigra and ventral tegmental area) [78]. This means that the midbrain regulatory influences, mediated by the DA control of tonic activation, exert inhibitory control on the limbic and neocortical networks of the cerebral hemispheres through collothalamic projections and striatal control that are closely regulated by ventral limbic activity [79].

A different form of subcortical control comes from the lemnothalamic pathway originating in the pontine brainstem (bypassing the midbrain) and targeting the dorsal limbic division, including the locus coeruleus norepinephrine control that is integral to phasic arousal [30]. This control system generates an excitatory, feedforward form of control, associated with elation [30], regulating the dorsal division of the limbic system. As Nauta [79] pointed out, the hippocampus (the base of the Papez circuit) provides important input to the nucleus accumbens of the basal ganglia, immediately adjacent to the septum (the other end of the Papez circuit). The nucleus accumbens is a key subpallial control structure for approach behavior [80,81].

Thus, the evolution of the mammalian cortex and limbic system speaks to a complex weave of excitatory, feedforward control (the foundation of the primitive pallium) and inhibitory feedback control (rooted in the subpallial striatum). As we attempt to characterize the mechanisms of active inference in the mammalian neocortex, both for perception [25] and for action [26], we need to understand how inhibitory elements from the embryologic subpallial ganglionic eminence, which migrate into layer 4 of the neocortex in mammals, are integrated with the foundational excitatory pallial architecture of the mammalian 6-layered cerebral cortex [30,31]. Fortunately, the challenging complexity of this evolved (and controversial) anatomy is less daunting when considering the more elementary separation of excitatory and inhibitory control modes in an evolutionary-developmental analysis of ancestral vertebrates.

### 3.6. Excitatory Pallial and Inhibitory Subpallial Origins of the Human Brain

**Figure 6:**
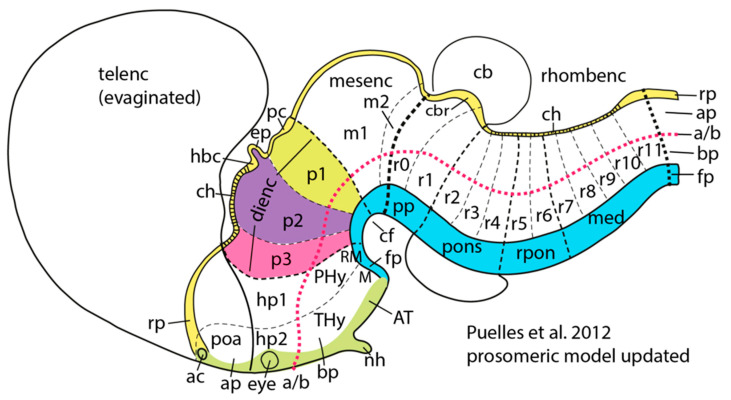
(Modified from Puelles [82]: Diagram of the prosomeric neuromeric model of Puelles and Rubenstein. The floor plate (fp; blue) is now defined molecularly and experimentally (it is induced by the notochord) as ending rostrally at the mamillary body (now understood to be a rostral entity). The roof plate (rp, yellow) is defined by experimental fate mapping as ending at the anterior commissure (ac) above the preoptic area (poa/ap). The red axial dash-line, similar to that in B, is the molecularly defined (Shh and Nkx2.2 markers) alar-basal boundary (a/b), not being referred expressly to a ventricular sulcus. The rostralmost prosomere is that identified as hp2, and its green-marked rostral limit extending between floor and roof plates is the acroterminal rostralmost domain of the brain (AT), where the rostral ends of the parallel floor (fp), basal (bp), alar (ap) and roof (rp) plates lie, and where the paired lateral walls of the tube (bp, ap) are continuous left to right. The telencephalon is interpreted as a bilateral dorsal alar evagination from the hypothalamic hp1 prosomere.

New insights into the evolutionary-developmental basis of mammalian neuroanatomy are being developed from studying the gene expression database of the Allen Institute [82]. Figure 6 shows that the major divisions of the neural tube, including roof, alar, basal, and floor plates, terminate at the hypothalamus, at the acroterminal domain. The entire telencephalon is then derived from the alar plate, responsible for the viscerosensory domain of the neural tube, specifically from the hp1 hypothalamic domain. What are the implications of this recent (in the time frame of evolution) mutation of genomic instructions that generate the vertebrate telencephalon from the alar plate?

The whole telencephalon appears to rest on a viscerosensory alar function. What does this imply for the continuing neural development of each individual? Perhaps the entire viscerosensory telencephalon is a kind of feeling-state: an experiential organ for higher vertebrate functioning. It imbues sense-making with a sentience by extending the basic (interoceptive) affordances of the neural tube grounded in the homeostatic visceral controls of the hypothalamus (prosomeres h1 and h2).

The hypothalamus is the end of the primordial neural tube. Even as the foundation of the vertebrate forebrain, the hypothalamus has always been regulated by the lemnothalamic projections conveying LC NE control (for mammals this is elation) as well as the VTA dopaminergic D2 collothalamic control (mammalian anxiety).

However, the telencephalon differentiated its sensory and motor capacities. Its initial organization in tetrapods (amphibians and reptiles) included a near-complete division between the excitatory pallium (pyramidal, glutamatergic) and the inhibitory sub-pallium [83]. The excitatory–inhibitory division was near-complete because within the pallium there were some inhibitory interneurons, and within the sub-pallium there were excitatory cholinergic cells, not unlike interneurons in the pallium.

**Figure 7:**
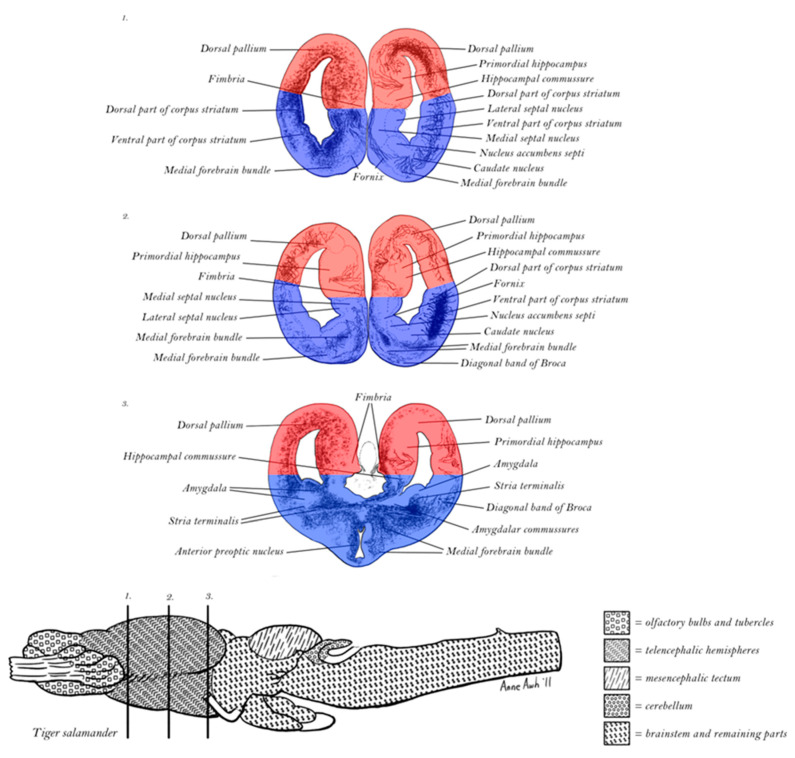
Approximate illustration of dorsal pallial (red) and ventral subpallial (blue) regions of the salamander telencephalon (original from Herrick, 1948), with approximate location of Herrick’s sections at bottom, and legend for major levels of the neuraxis.

With these important complexities, we see a remarkable pattern of **E**–**I** separation in the ancestral organization of the telencephalon as shown in Figure 7. In the elaboration of hp1 to form the telencephalon, the excitatory control of the pallium was fully separated from the inhibitory control of the sub-pallium (striatum). Each had its own input from the lower brain that remains integral to the subcortical control of active affordance in mammals [30]. Simplifying a complex story, we can see how the basis for the excitatory pallium is formed by the lemnothalamic projections from the pontine brainstem; the basis for the inhibitory sub-pallium is formed by the collothalamic projections from the midbrain.

Understanding these origins helps to interpret the adaptive evolution of the mammalian cortex after the invasion of the pallium by subpallial elements [42,84,85]. Although the specifics are debated, there was apparently a large embryologic mutation in which inhibitory interneurons migrated from the ganglionic eminence into the cerebral cortex, providing additional inhibitory control for layer 4 that was important to the differentiation of the 6-layered cerebral cortex [42,84,85]. The excitatory and inhibitory relations took on a new form, that of the cortical columns. These became the integrated processors for the cerebral cortex of mammals [32].

Thus the evolutionary history of the telencephalon points to the fundamental division between excitatory pallial and inhibitory subpallial forms of control, and understanding these origins may help to explain the integration of control systems that underwrite the excitatory predictive control of the dorsal limbic division, balanced by the inhibitory corrective control of the ventral limbic division and striatum [30].

Even with the extensive weaving of excitatory and inhibitory controls within the mammalian cerebral cortex, there is another clear separation of these fundamental forms of neural control. This is in the differential forms of memory consolidation in the NREM and REM stages of sleep.

## 4. Inhibitory and Excitatory Neurophysiology of Memory Consolidation in Sleep

The memory capacities—that are realized by **E** and **I** control of conscious cognition—may be emergent from the sequential neuromodulatory control of REM and NREM stages of sleep, respectively. In reviewing these mechanisms, Tucker, Luu, & Friston [33] began with the remarkable observations suggesting that explicit memory, the ability for declarative recall of specific facts, has been shown to be consolidated in NREM sleep, but not REM [86,87,88]. More specifically, the ability to discern causal relations among elements in certain problem-solving tasks has been shown to improve more under sleep, and particularly NREM-rich sleep, more than an equivalent period of waking [86]. An important interpretation is that the replay and rumination of events (observed primarily through neural activity recorded in NREM sleep) may allow this installation of causal associations via synaptic consolidation [86].

Clearly, each of the five or so cycles of human sleep involve both NREM episodes, which are the entry point to sleep in the early night, as well as REM bouts, which are brief in early cycles but increase in later cycles of the night. Yet memory testing has confirmed the dependence of explicit, declarative memory on NREM—to the point that two of the major scientific reviews of this literature (focused on experimental evidence) have commented that it is difficult to understand the purpose of REM.

Evidence speaks to the importance of REM for emotional memory, and emotional adjustment [87,88,89]. Furthermore, several findings suggest the importance of REM to *implicit* memory, including the ability to understand the background context that often allows solutions to insight problems [86,87,89]. Once we formulate the mechanisms of excitatory as well as inhibitory consolidation of synaptic organization in memory, it should become clear that REM is as integral as NREM to adaptive self-organization of the synaptic connectional architecture.

### 4.1. Inhibitory Specification and Error-Correction in Explicit Memory

In reviewing the neurophysiological evidence on memory consolidation in sleep, Tucker, Luu, and Friston [33] proposed that the dorsal and ventral limbic divisions are consolidated in REM and NREM stages of sleep, respectively. The excitatory feedforward regulation of the dorsal limbic division, under control from the pontine reticular activating system and its lemnothalamic projections, supports the generation of predictions in the limbifugal direction (from limbic toward neocortical areas in the Structural Model). This excitatory, *feedforward* (top-down) control process appears to be consolidated in the excitatory (cholinergic) neurophysiological regulation of cerebral networks in REM sleep.

Similarly, the inhibitory feedback (bottom up) regulation of the ventral limbic division supports the error-correction required for unpredicted events in the limbipetal direction of predictive processing, which appears to be consolidated in the inhibitory (largely GABAergic) mechanisms of NREM sleep.

With these separate mechanisms, sleep consolidates predictive processing in reverse. The entry point to normal sleep is invariably NREM, which consolidates the accumulated errors (unpredicted events) experienced during waking. The neurophysiology for this encoding of explicit memory for unpredicted events is largely inhibitory. The replay of significant experienced events is engaged through claustrum suppression of excitability in the hippocampus, generating sharp wave ripples that index the unpredicted events [90]. These sharp waves are grouped and encoded by sleep spindles regulated by the GABAergic (inhibitory) control from the Thalamic Reticular Nucleus (TRN). Coordination of the spindle encoding—and protection from further plasticity—is organized by the widespread inhibitory control from Slow Oscillations (SO), regulated by projections from the claustrum [91]. Tucker et al. [33] proposed that the inhibitory delineation of unpredicted salient events—through these mechanisms—is the basis for consolidating explicit memory; in ways that allow the feedback (error-correction) phase of predictive processing to incorporate unpredicted events within the organism’s (generative) model of the world. NREM sleep is thus not just an encoding of memory, but a continuation of active inference.

Note that the dominance of inhibitory control in NREM sleep has the straightforward effect of renormalizing neuronal activity, thereby providing a mechanism for synaptic homeostasis [88,92]. Instead of a generic normalization, however, NREM memory consolidation first engages inhibitory mechanisms to select and delineate recent salient (i.e., unpredictable) events—including their causal relations—so they are retained and stabilized by the subsequent global synaptic normalization of the widespread SOs.

### 4.2. Excitatory Reconsolidation of the Bayesian Predictions of Implicit Memory

As important as it is to delineate and consolidate unpredicted events, the implication is that new information will disrupt representations of prior memory, due to the inherent stability-plasticity dilemma of distributed memory systems [93]. Tucker et al. [33] propose that the subsequent process of REM sleep serves to reorganize, and thus reconsolidate, existing organismic memory (that underwrite Bayesian priors) in light of the new NREM encoding of unpredicted events.

The neurophysiology of REM sleep includes widespread cholinergic excitatory modulation of the cerebral networks, including both the pedunculopontine modulation of thalamocortical regulation of cortical excitability (a primary source of the “paradoxical” EEG activation) and the widespread forebrain NB ACh control (Figure 2). Several lines of evidence point to the excitatory control being attributable to the lemnothalamic regulation of the dorsal limbic system particularly. This evidence includes the activation of immediate early genes in the dorsal limbic division following the intense REM rebound after REM deprivation [94]. Other key evidence is the regulation of the theta oscillations of REM by multiple levels of the pontine lemnothalamic pathway [95,96].

How can we understand the reorganization of existing memory by widespread excitatory activation in REM? Tucker et al. drew on modeling by Hinton and associates with a sleep–wake algorithm [97]. When a connectionist simulation is allowed to “run free,” without the constraint to fit the predictions to sensory input, the effect is to reorganize the existing connection (prediction) weights in a more efficient form. This is particularly effective when the excitatory exercise is constrained to maintain minimal complexity or description length, and thus to minimize variational free energy. The result is a *gist reconstruction* of the internal prediction weights, an efficient (and schematic) reorganization of the Bayesian priors for the system’s generalization to new events on reawakening.

### 4.3. The Unconscious Sources of Waking Consciousness

Considering the integral role of sleep consolidation in the adaptive control of cognition, we can see how conscious processing may involve a complementarity of **E** and **I** control, prepared for each day’s experience by the previous night’s sleep. More than just consolidation, the neurophysiology of sleep refines the organism’s cognitive model of the world, starting with the critical and corrective mode—organized by the subcritical inhibitory state of NREM sleep—and then progressing in the next stage of sleep to the generative, imaginative, predictive mode that has been reorganized by the supercritical excitability of REM sleep [33].

The neurophysiological underpinnings of consciousness can then be understood as the regulation of neural networks by the primordial neural control systems, excitatory phasic arousal (**E**) and inhibitory tonic activation (**I**). In this way, waking consciousness emerges from our feelings, tuning in real time to criticality by degrees of excitatory elation and inhibitory anxiety.

Remarkably, it is the extreme variations, from inhibitory consolidation in NREM to the excitatory organization in REM, which consolidate their influences on memory in sleep. The neurophysiological control of synaptic homeostasis [46,88] could then be reformulated as more than a synaptic process, and rather as an adaptive organismic process, accumulating homeostatic controls from the waking excursions of **E** and **I** not only to renormalize but to realize their cognitive differentiation and integration in sleep.

## 5. The Criticality Hypothesis of Consciousness

With this background—on the foundations of excitatory and inhibitory control in the dual memory systems—we can formulate a neurocomputational hypothesis to suggest how subcortical mechanisms of waking arousal, with distinct qualities of feeling, organize the dual limbic forms of working memory that—through their balanced interaction—generate consciousness. The subcortical arousal controls—characterized as the pontine-lemnothalamic phasic arousal for excitation (elation) and the midbrain-collothalamic tonic activation for organismic inhibition (anxiety)—are the adaptive regulators of the dual memory systems. These complementary control systems regulate consolidation each night and then regulate active inference and experience during waking.

### 5.1. Excitatory–Inhibitory Balance and Criticality

Excitatory–inhibitory (**E**–**I**) balance is essential to organizing neural networks at multiple levels of the human nervous system [98]. This aspect of neuronal self-organization can be complicated, such as in networks where an excitatory glutamatergic projection has an inhibitory influence at a systemic level. An example is how the claustrum’s excitatory projections to the cortex terminate on GABAergic interneurons that then exert a powerful net inhibitory effect [91]. Acknowledging these complexities, we can see how **E**–**I** balanced networks provide a cybernetic mechanism for constructing multiple levels of stable and yet adaptive control in neural networks.

In our effort to align the neurodevelopmental process of progressive anatomical-connectional self-organization with the Bayesian belief updating of active inference, we have suggested that the excitatory control of the dorsal limbic division is the motive control for the cortex’s generation of predictions, whereas the inhibitory control of the ventral limbic division underlies error-correction [30,32]. One might ask how the organization of complex information constructs—concepts and experiences in the attractor dynamics of cell assemblies —inherit from the dynamics of network control in the brain.

### 5.2. Criticality in Brain Systems

In mathematical models of complex systems, there are remarkable increases in the complexity of causal interactions at the point of *criticality*, exactly between two phases of the system, such as order versus disorder [99]. Whereas the control properties are quasi-linear at a distance from criticality, they become more complicated closer to criticality (e.g., exact **E**–**I** balance), where high-order terms can no longer be ignored. Intuitively, small adjustments in **E** can be accommodated by small excursions in **I** in ways that are easily manifest in hierarchically rich patterns that are not possible when either **E** or **I** dominate. The result of **E**–**I** balance is a regime of cybernetic sensitivity that allows complex control of a system when this balance is maintained [100].

Computational models of neural networks often show this exquisite sensitivity to control parameters, as well as a wide dynamic range in responding to inputs, specifically in the regime of control criticality [101]. However, developing measures of criticality has been challenging. The clearest in vivo models have been neuronal avalanches [102], in which the **E**–**I** balance determines optimal neural propagation (avalanche at 1/N where N = number of target connections). At this point of optimality (stable rate of activity spread), neural firing propagates through an ordered avalanche across the network: too low and it stops; too high and it diverges exponentially out of control into a seizure. Convincing early demonstrations have used large neural network simulations, in which simulated neurons operate most efficiently in a large ensemble, with wide dynamic range, when maintained at, or near, a critical balance [103].

### 5.3. Criticality at the Edge of Active Inference

Mathematical formulations of criticality bear a close parallel to the Bayesian applications of the free energy principle to active inference in the nervous system [23]. As shown in Figure 4, the neural mechanisms of auditory perception begin with predictions of what will be heard, and these predictions are updated (error-corrected) by the sensory information. In unpacking this the gradient descent on free energy—using a model of bird song perception—Friston et al. demonstrated the emergence of criticality, as a slowing of the dynamics within a regime of instability, which is exactly aligned with minimizing free energy (Figure 9 in Friston et al. [23]).

If we are correct in associating the motive control of prior predictions with the feedforward bias of the dorsal limbic division, complemented by the inhibitory control of error-correction by the ventral limbic division and striatum [30,31], then there may be a similar criticality of limbic control that is approached at the cutting edge (of chaos) in active inference. This criticality emerges at the interface or surface where Bayesian *priors* meet the error-correction *likelihood* of sensory evidence. For each perceptual, motor, and cognitive operation, this ‘edge’ defines a Markov blanket [104,105]; namely, the boundary between the intrinsic information of the self (the subjective Bayesian priors of private knowledge) and the extrinsic information from the world (i.e., objective sensory-motor constraints for reality-testing against observations).

### 5.4. Synchronizing Vertical Integration

If we are to anchor this model in neuroanatomy, achieving this balance in corticolimbic control would require criticality throughout the vertical neuraxis. The neocortical networks of the Structural Model—and the multiple Markov blankets at each hierarchical interface—must be tuned by limbic excitatory (dorsal) and inhibitory (ventral) regulation. Moreover, these limbic controls are fully dependent on their subcortical arousal and activation controls, including the lemnothalamic pontine NE and 5HT neuromodulators of phasic arousal, as well as the midbrain DA and pedunculopontine cholinergic (PPT ACh) neuromodulators of tonic activation [73,75]. This dependency requires that self-regulation of criticality—in neocortical processes of prediction and correction—achieves a long-range synchronization of the major control structures of the neuraxis (Figure 2).

To speculate on the dynamic control for this vertical (neocortical-limbic-subcortical-spinal) integration, we point to the phenomenon in criticality described as *scale–invariant correlation* [99]. The brain seems to have evolved such that its excitatory controls achieve the exact balance with its inhibitory controls as they gain a dynamic (necessarily latency-adjusted) scale–invariant correlation across the multiple levels of control in the vertical neuraxis, from the spinal cord to the frontal lobe.

By drawing on the control efficiency achieved by excitatory and inhibitory mechanisms operating in balance at criticality, our hypothesis is that the motive controls regulating the excitatory generation of prior predictions exactly balance the inhibitory feedback constraint of error-correction at each level of the vertical neuraxis. Importantly, the Bayesian formulation shows this clear range of criticality dynamics—revealed by the sensitive instability (i.e., sensitivity) induced by control parameters—where the descending prediction of the intrinsic information meets sensory evidence from the world in the (ascending) feedback direction [23].

Thus, reasoning from how these modeling results align with the foundational principles of vertebrate neuroanatomy, we propose that the balance between impulse and constraint [42] is indeed an excitatory–inhibitory balance (in the control of the dual limbic memory systems). Criticality then becomes an *organismic* control state, where these modes of self-regulation achieve effective control dynamics for active inference in waking cognition.

This is how the control of the neurocomputational process of consciousness (active inference) is regulated by our feelings. To be fully sentient, we must balance the limbic control of excitement (feedforward limbifugal control of elation) with that of inhibition (feedback limbipetal constraint of anxiety) in an exact and precisely regulated balance to achieve the requisite state of criticality. The adaptive control of criticality then supports hierarchical belief updating, and the metacognitive capacity for reflective awareness, that we experience as conscious experience. On this view, maintaining criticality rests upon deploying modulatory control that can be read as mental action that, in psychological terms, speaks to sensory attenuation and selective attention.

### 5.5. Brain Criticality and Its Variations

The underlying neurophysiology of these dual memory control systems may be revealed by recognizing that they have evolved to be consolidated separately in REM and NREM sleep [106]. The waking cognition, described as active inference, may be optimized by a closely regulated balance between the excitatory limbic feedforward control of descending predictions, consolidated each night in REM, and the inhibitory limbic feedback control of ascending corrections (prediction errors), consolidated each night in NREM.

Practically, the self-organized criticality of conscious processing is a delicate state to maintain, as William James observed. More importantly, we need to become more excited when opportunities arise (following what James described as his felt furtherances) and attenuated when encountering threats (felt hindrances). As a result, the criticality of consciousness appears to have evolved as a *critical variations*, naturally following a process of Bayesian inference under uncertainty: Processing shifts between the differing requirements for prediction (augmenting Bayesian priors to attenuate sensory evidence) and correction (attenuating Bayesian priors to augment feedback constraints supplied by sensory evidence). These requirements vary continuously in a capricious world depending upon the relative confidence (i.e., precision) afforded by beliefs, relative to sensory evidence. In a volatile (threat laden) world, greater precision or weight should be afforded sensory evidence (e.g., in the form of precision-weighted prediction errors). Conversely, when in a predictable scenario, more weight should be afforded to prior beliefs and their accompanying predictions. However, in both cases, balance has to be maintained for optimal belief updating.

Because waking consciousness requires self-organized criticality (being awake enough to pay attention), the full uncoupling of these adaptive control mechanisms may only be possible in the unconscious stages of sleep. In sleep, the entire cerebral network architecture can be consolidated first by the subcritical, inhibitory state of NREM and then by the supercritical excitatory state of REM sleep. This conclusion is consistent with Hering’s [107] formulation that—because consciousness requires ongoing support from the mechanisms of memory—memory organization (consolidation) can only occur in the unconscious workings of sleep [86].

As a result, the nightly outcomes of sleep are what set the stage for the balanced excitatory and inhibitory controls on working memory that support active inference in waking. In short, the most effective states of active inference and conscious awareness arise when these control systems are balanced. In what follows, we provide a formal account of criticality of this narrative.

Section 6 introduces the control parameters (excitation **E** and inhibition **I**) that undergird criticality, using the formulation of variational free energy as complexity minus accuracy [105]. The excitatory influence of elation enhances confidence in the prior beliefs and provides more precise contextual guidance for hierarchical predictive processing. In contrast, the inhibitory influence of anxiety increases prior uncertainty, thereby emphasizing the precision or influence of (corrective) sensory evidence. Section 7 presents a summary of the criticality hypothesis, in which the dynamics of precision are regulated by our feelings, and the ensuing free energy determines the felt breadth and stability of conscious experience. Section 8 then extends the criticality hypothesis to include the homeostatic regulation of excitation and inhibition in sleep, in which excursions of **E** and **I** far from criticality admit major forms of network reorganization that set the nightly stage for the next morning’s ignition of conscious experience in regimes of self-organized criticality.

## 6. Excitatory Control of Complexity and Inhibitory Control of Accuracy in Active Inference

### 6.1. Terms for Formulating the E–I Gain Control of Active Inference

In this section we provide a systematic statement of the limbic control of excitation and inhibition in terms of gain control (Table 2). 

### 6.2. Overview of Active Inference and Neuronal Belief Updating

Active inference can be expressed as minimizing variational free energy (F) defined as **complexity** minus **accuracy** [105]. In this Bayesian formulation, the brain estimates the causal states of the world (s) given the sensory observations (o), under a generative model. A generative model is specified in terms of a likelihood and prior: e.g., p(o,s) = p(o|s)p(s). Inversion of this model leads to an estimation of latent or hidden states in terms of a (variational) posterior q(s), that minimizes (F):

F(q, o) = D_KL_[q(s)||p(s)] − E_q_[ln p(o|s)]                      = E_q_[ln q(s) − ln p(s)] − E_q_[ln p(o|s)] (1)

- The first term on the right is named ***complexity***: it measures how far the posterior solution q(s) deviates (by the Kullback–Leibler divergence, D_KL_) from the original prior estimate p(s). This term minimizes free energy by keeping the updated beliefs q(s) close to the priors p(s). In other words, it scores the degree to which observations “change one’s beliefs.”
- The second term on the right is ***accuracy***, defined as the expected likelihood that the posterior beliefs (q) successfully predict the data o. A more accurate posterior belief solution minimizes the fit error (e.g., precision-weighted prediction error) and thus minimizes F.

Active inference can then be seen as finding the most likely causes of observations that remain consistent with prior experience (i.e., the world is as you know it) yet is maximally accurate in explaining the observed evidence. The active part of active inference can only change observable outcomes and therefore can only change the accuracy through action; however, here, we will be primarily concerned with perceptual inference.

### 6.3. Introducing the E–I Control Gains

The criticality hypothesis introduces two (affective) gain control or precision parameters based on the literature on limbic regulation of cognition, feedforward excitation (**E**) and feedback inhibition (**I**) that determine how strongly each term (complexity or accuracy) influences variational free energy. This formulation is based on the association of prior precision (complexity) with the (excitatory) dorsal limbic division and the precision of sensory constraints (accuracy) with the (inhibitory) ventral limbic division:

F(q, o) = D_KL_[q(s)q(**E**)q(**I**)||p(s, **E**, **I**)] − E_q_[lnp(o|s, **I**)] = E_q_[ln q(s)q(**E**)q(**I**) − ln **p**(s)^E^ − ln p(**E**,**I**)] − E_q_[ln**p**(o|s)^I^] = E_q_[ln q(s) − E·ln **p**(s)] + D_KL_[q(**E**)q(**I**)||p(**E**,**I**)] − E_q_ [**I**·ln**p**(o|s)] (2)

The excitatory control **E** can be thought of as enhanced **confidence** or precision that biases posterior beliefs towards prior predictions. The inhibitory control **I** can be thought of as a complementary **vigilance** that is associated with an increased precision of the likelihood—that accompanies anxiety—which biases belief updating towards fitting the sensory evidence or observations. Note that **E** and **I** are simply scaling constants. Technically, they play the role of precision, where baseline priors and likelihoods (in **bold**) have a Gibb’s form. Their expected values **E** and **I** then minimize variational free energy in a way that depends on the functional form of the generative model:

**E** = arg min F(q(s)q**_E_**(**E**)q(**I**), o)    **I** = arg min F(q(s)q(**E**)q**_I_**(**I**), o) (3)

Please see Parr et al. for a detailed description for generative models based upon partially observed Markov decision processes—and link to classical neuromodulators [44]. An equivalent treatment of continuous state space models can be found in the literature of variational Laplace [108]. For simplicity, we have ignored posterior beliefs about policies or action in this basic formalism.

With this basic formalism in place, we can now consider qualitative consequences of optimizing **E** and **I**, according to Equation (3), including conceptual coherence and temporal persistence, via their effects on complexity and accuracy.

### 6.4. How E and I Regulate Cerebral Networks

Table 3 summarizes how the E and I factors of gain control operate to modulate the precision factors for the priors and sensory error-correction in active inference.

Equation (2) is the complexity–accuracy formulation of active inference with complexity (**E**) and precision (**I**) gains applied to the two components of the generative model; namely, the priors and likelihoods, respectively. Neuromodulatory systems are generally thought to control these gains, cycling brain dynamics through excitatory and inhibitory regimes, while ensuring criticality—in the sense of maintaining the right balance, so that complexity constraints are never lost while, at the same time, observations continue to inform belief updating. Criticality is assured because variational free energy is minimized, following the arguments in [23].

## 7. Criticality of Consciousness 

Table 3 considers the various aspects of excitation–inhibition (**E**–**I**) balance in belief updating.

### 7.1. Precision Dynamics of Feelings

Elation (**E**) accompanies increases in prior precision, subjectively inner confidence; conversely, anxiety (**I**) reduces the contribution of prior constraints by increasing the precision of information conveyed by observations. Equivalently, anxiety sharpens vigilance (sensory precision) to incoming prediction errors (enhancing NREM sleep pressure); while elation blunts their ability to update posterior beliefs (enhancing REM sleep pressure).

### 7.2. Experience Bandwidth and Stability

Important effects of affective arousal on cognition and experience are the changing conceptual scope of posterior representations and the temporal persistence of belief states. Elation increases confidence and the scope of prior beliefs to explain (multimodal) sensory evidence at hand. Ideation is expansive, holistic, and integrated. But this is at the expense of sensitivity to evidence (e.g., prediction errors) and the temporal persistence of ensuing belief updates. Anxiety narrows the associational cohesion afforded by precise priors, such that ideation is externally focused and differentiated, which is accompanied by enhanced temporal persistence of responses to sensory input. The nature of the belief updating is subject to fluctuations (flightings and perchings), creating a kind of criticality of conceptual differentiation and integration, determining whether we are focused, expansive, or somewhere in between.

Anxiety (**I**) and elation (**E**) direct attention to the sensorium and prior narratives, respectively. Anxiety creates tonic activation and James’s perchings of consciousness. Elation creates phasic arousal and orienting responses associated with James’s flightings of consciousness. In either imbalanced state, **E** > **I** or **E** < **I**, we are affectively biased and thus motivated in consistent ways (approaching or avoiding, respectively). Without adequate sleep homeostasis, criticality may be elusive, and psychopathology may ensue. This follows because sleep homeostasis is necessary to update the baseline priors that underwrite complexity and its contribution to variational free energy.

### 7.3. Variational Free-Energy

Variational free energy is minimized as prediction accuracy increases for both perchings and flightings of conscious experience. Anxiety speeds online correction; elation speeds offline hypothesis consolidation. The statistical physics of control theory and the correlates of criticality (e.g., critical slowing) suggest that experience has a variable extent in time. Moreover, the exciting and inhibiting arousal control systems—our feelings of furtherance or hindrance—structure this extent in predictable ways. In this regard, it is useful to note that precision (i.e., control gain) plays the role of a rate constant that controls the rate of belief updating and therefore the passage of subjective time as measured by information rate [109].

With imprecise predictions, we cannot sense the future. And the past ceases to be informative. In this sense, increased sensory precision (**I**) tips the balance of evidence accumulation towards sensory evidence and away from prior predictions, meaning that we update our beliefs more quickly with less constraints from the past. Criticality expands the experience of time to allow active inference to optimally integrate objective (sensory) evidence and the predictions of subjective opportunity. Criticality is the broad zone for stability and plasticity in the balanced moment. Yet balance is not enough: complexity and accuracy are both necessary to minimize free energy and thus optimize the span of consciousness.

### 7.4. Criticality Condition

Conscious flow feels vivid yet coherent when ρ stays near its optimum and we are more or less in **E**–**I** balance. As we build up sleep debt during the day, we have the homeostatic motivation to re-balance the memory systems during the nightly exercise of NREM-REM memory consolidation. Then, after progressively self-organizing through the nightly cycles, the **E**–**I** balance upon wakening is prepared more or less at criticality, so we wake up more or less conscious. Criticality then allows for increased order in experience. When both anxiety (differentiation) and elation (integration) are balanced and hierarchically integrated [3,110], we achieve increased conceptual structure and information order (↓ F) at the critical edge of active inference [23].

In contrast, when we are off-criticality, we are biased toward one control, **E** or **I**, more than the other. This, of course, is necessary to become motivated (some combination of elation feedforward impulse to approach or anxiety feedback constraint to avoid). The variational cybernetics must be adaptive, responding to requirements for life. We self-regulate to be differentiated when we have to make discriminations or integrated when we need to see the big picture, thereby varying both conceptual structure and temporal stability in degrees through variational criticality.

**Phenomenological Summary:** Elation widens conceptual scope, reduces the rate of belief updating, boosts inner confidence (ρ ↑) and blunts sensory vigilance (ρ ↓). Anxiety augments sensory attention (ρ ↓), lowers self-certainty (ρ ↑) yet sharpens feedback reliance. Each of these arousal systems (**E** and **I**) are consciousness flow regulators. Their continuous opposition nudges the system toward a moving, albeit often turbulent, window of criticality where subjective experience feels both rich and differentiated, and yet coherent and integrated at the same time.

## 8. Variational Dynamics of Sleep Pressure in Waking Consciousness 

**Figure 8:**
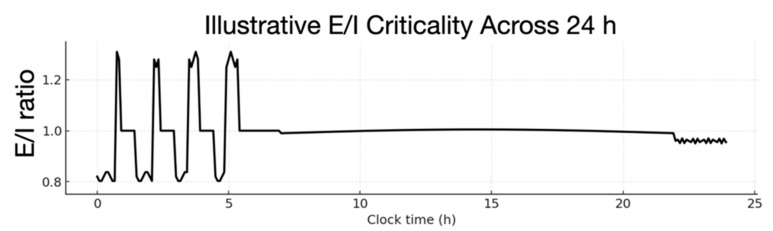
Circadian variation in criticality, illustrated by a simple model that implements NREM and REM pressures to create the sleep stages, and criticality during consciousness, by tuning the ratio ρ = **E**/**I**. The NREM stages trigger excursions to subcritical levels, and the REM bursts trigger supercriticality.

Figure 8 illustrates the circadian variation in **E**–**I** criticality. The sleep pressure of REM (C process) is regulated over 24 h. by the coupled mechanisms of orexin and MCH (melanin concentrating hormone). NREM pressure builds with the waking accumulation of adenosine, and NREM suppresses arousal controls via the ventrolateral preoptic area (VLPO). There is a marked drop to subcritical range with each NREM episode, and a phasic excursion to supercriticality with multiple PGO discharges during each REM episode. Fortunately, the avalanche propagation does not become supercritical (thereby avoiding seizures) during REM sleep, even though the excitability of highly diverse associations (the **E** parameter) is extreme. The ρ = **E**/**I** ratio plummets in each NREM interval, as inhibitory control selectively differentiates and stabilizes the cerebral networks.

### 8.1. Memory and Cognition Consolidation

The neurophysiological mechanisms of NREM and REM sleep serve as consolidation mechanisms, operating to continue the process of active inference through inhibitory specification of explicit memory in NREM and excitatory exercise of predictive capacity in the context of the organism’s prior knowledge (Table 4).

Sleep therefore operates as a limit-cycle controller: NREM drains inhibitory pressure, REM drains excitatory pressure, and their alternation returns precision variables to the critical window needed for optimal consciousness for waking the next morning. Across the night, each stage implements a distinct precision operation on centrifugal (top-down priors: **E**) and centripetal (bottom-up corrections **I**), constraints, letting the brain uncover causal relations that were implicit—but unrecognized—during wake. The sleeping mind processes information through an inverted (differentiate and correct first in NREM, then integrate and prepare to predict in REM) mode of active inference, in the absence of sensory afference [33].

### 8.2. Balance of Adaptive Controls in the Stages of Sleep

The consolidation of memory is not a simple recording process, but a two-stage organizational process through which inhibitory neurophysiology (hippocampal sharp wave ripples, spindles, and slow oscillations) in NREM sleep and the excitatory neurophysiology of REM sleep (global cholinergic excitatory exercise of predictive priors in the absence of sensory-motor contraints) sequentially organize both cognitive capacity and phenomenal experience (Table 5).

Thus, NREM differentiates experiential components (objects) and encodes reliable temporal order, while REM performs creative, holistic counterfactual exploration on that scaffold. Their alternation tunes the underlying neurophysiological drivers of **E** and **I**, so that waking network criticality begins the next day near its optimal corridor.

Under this account, the diurnal cycle of REM and NREM homeostasis changes their balance during the waking consciousness of the day. The diurnal orexin/MCH cycle leaves REM pressure high in the morning, such that the consciousness of the morning is weighted toward **E** and integrative cognition. As NREM pressure builds in the afternoon due to gradual adenosine build-up, the balance shifts toward **I**, leading to improved attentional focus and cognitive routinization for detailed tasks.

## 9. The Criticality of Consciousness and Its Variational Control

The important evidence base for understanding consciousness may be the neurophysiology of memory consolidation in sleep. What is described in cognitive neuroscience as memory consolidation [42], appears to involve inhibitory mechanisms (NREM) alternating with excitatory mechanisms (REM), each of which contextualizes the brain’s process of correcting its actions and organizing its anticipation of future opportunities.

As we follow the implications of these dual modes of self-regulation into waking cognition, the first question is the transition from alternation in the stages of sleep to the simultaneous operation of these dual control systems in waking cognition. The hypothesis is that consciousness provides expanded information capacity through **E**–**I** criticality with a balance of the excitatory and inhibitory regulatory (memory and arousal) systems.

A second question is how the dynamic balance of these controls regulates ongoing cognition and behavior, given their motive biases; varying from depression to elation for the phasic arousal system and from security to anxiety for the tonic activation system. This question is addressed by the hypothesis that consciousness is regulated adaptively—through excursions between the phasic arousal of elated excitement (**E**) and the tonic activation of anxious inhibition (**I**)—in the adaptive criticality of Bayesian mechanics.

For both these operations, the consolidation of the dual-motive-memory systems in sleep prepares them to support the cognitive operations of consciousness. Furthermore, the motivational properties of the excitatory dorsal limbic division and inhibitory ventral limbic division and striatum provide important clues to the structural changes in working memory associated with criticality.

The dialectical balance between the dorsal limbic impulse (**E**) and ventral limbic constraint (**I**) in waking cognition provides the adaptive control of structure (conceptual scope) and process (temporal persistence) that when optimally tuned admit the expanded scope of metacognition that we experience as waking consciousness. By appreciating the dual NREM and REM memory systems—through observing their unique forms of inhibitory and excitatory neurophysiology in sleep—we may gain insight into the ongoing forms of neuromodulatory control, including intrinsic motivational and affective properties, that support memory access in waking consciousness. The exact balance of the dual memory systems and their inherent adaptive controls may be essential to the expanded explanatory scope of **E**–**I** criticality in consciousness. The controlled variations in **E**–**I** balance in waking may then allow the dynamic operations of criticality to extend further into the fringes of implicit prior predictions (**E**) and explicit environmental constraints (**I**) that are essential to active inference.

Although the adaptive variations toward greater **E** or **I**—according to situational demands—are integral to our hypothesis, the central phenomenon is the expansion of information processing capacity that is achieved at the exact **E**–**I** balance. This is where we propose that both the functional utility and the phenomenology of human consciousness may arise.

### 9.1. Spanning the Specious Present

Integral to both effective cognition and experienced phenomenology is the continuity of experience in time. William James emphasized that we must apprehend some considerable duration in the stream of consciousness; otherwise, experience is reduced to the *specious present* [7]. With no residual continuity of past memory, and no sense of the future, the “present” of consciousness collapses to a point with no duration. Certainly, the concept of working memory, or a global workspace [111], implies that there is some continuity of cognitive operations in time. We propose that this emerges from (waking) contributions of the dual memory systems operating at criticality.

A well-known marker of criticality in complex systems is the presence of *long range temporal correlations*: extended relations of network patterns in time that emerge with the appearance of criticality, such as we propose appear at exact **E**–**I** balance [99,101].

For example, in a recent demonstration of long range temporal correlations in cognitively functional states of human consciousness, Muller and associates [112] examined high frequency gamma oscillations (56 to 96 Hz) in the intracranial EEGs of patients with epilepsy, using the autocorrelation function to characterize long-range correlations in the propagation avalanches of high gamma, reflecting the regulation cortical activity at multiple temporal scales. Cognitive functioning in the conscious state was associated with the appearance of long-range correlations, whereas these autocorrelations were significantly shortened by disruptions of consciousness, such as by interictal epileptiform discharges, anti-seizure medications, or periods with slow-wave (sleep-like) activity.

Given the importance of empirical validation, the ability to measure the neurophysiology of cortical excitatory mechanisms (such as gamma and theta-gamma phase coupling) and inhibitory mechanisms (such as beta and alpha-beta phase coupling) in the EEG admits empirical predictions about the span of neural states in time that allow empirical tests of the criticality hypothesis.

Within the general regime of criticality, as described by the relations in Table 2 and Table 3, variations in **E** and **I** will shift the limited capacity of waking working memory in ways that determine how experience emerges from the specious present. This emergence can be understood as the access to the information—encoded by synaptic connectivity—experienced psychologically as mental associations. This access is parameterized by the neural control system parameters of conceptual scope as enhanced by **E** and temporal persistence as enhanced by **I**. These parameters reflect the **E**–**I** regulation of the dynamics of what we might describe as *corticolimbic resonance*, patterns of neural activity traversing the architecture of the Structural Model between the massive representational capacity of the neocortex, hippocampus and dorsal limbic division (**E**) on the one hand and the amygdala, striatum and ventral limbic division (**I**) on the other. These dual-motive-memory systems provide dual modes of regulating consciousness [15] adaptively [33].

Excitation has the simple effect of increasing synaptic efficacy and expanding the scope of associations enjoined by current consciousness. It is directly supported by the dorsal limbic controls, including theta oscillations (recruiting the full pontine lemnothalamic pathway that generates the PGO discharges of REM) and their recruitment of local gamma phase-aligned cell assemblies that support the broad conceptual scope and feedforward bias of the dorsal corticolimbic division. Due to the habituation bias of elation [73], there is a corresponding reduction in temporal persistence that causes the access to broad associations to be ephemeral.

Importantly, as formulated in Equation (1), balance toward **E** involves experiential and behavioral dominance by the predictive, self-evidencing Bayesian priors, the intrinsic domain of the self. The exaggerated elation (**E**) of clinical mania is thus associated with the *flight of ideas*, involving a failure of attention, and the untethering of belief updating from sensory constraints. At the same time, with enhanced conceptual scope, moderate degrees of mania (hypomania) lead to creative and expansive thinking [113,114]. When unbalanced by sufficient anxiety, exaggerated mania leads to pathological grandiosity, due to the narcissistic intrusion of the Bayesian priors of the self, without adequate constraint from inhibitory anxiety.

In contrast, exaggerated anxiety (**I**), particularly without balance by adequate elation, is associated with excessive focus and routinization of cognition, extending temporal persistence and emphasizing feedback control for enhanced accuracy (i.e., sensory precision). At the same time, there is a restriction of temporal scope that excludes future events from consciousness, particularly those predictions associated with personal confidence, such that uncertainty is maximal. Because the operation of prior predictions emerges from the REM-consolidated self [115], the inhibitory constraint of these priors in high anxiety is not only a constraint on creativity, but a constraint on self-evidencing [36].

Thus, the assimilation and accommodation of effective cognition involves a mode of information processing that is also a mode of self-regulation. As the **E**–**I** balance is regulated in the regime of criticality, the adaptive process of consciousness achieves both optimal cognitive control and optimal personality control when the self-regulation of feelings balances exactly at criticality.

### 9.2. Organizing Conceptual Complexity

Given the massive connections of the human brain, an important question is how the dual-motive-memory systems contribute to the *structure* of the currently active representations (percepts and concepts) that are organized and experienced in consciousness. We have proposed that the dual memory systems provide varying contributions to conceptual scope, or breadth of cognitive associations, during their dominance of NREM and REM sleep. A restricted scope and more differentiated structure support the delineation and articulation of unpredicted events in NREM. A greatly expanded associational scope (more holistic structure) allows the exercise of unbounded predictions in REM. These influences appear to remain important to the ongoing modulation of conceptual structure and thus cognitive organization in the criticality of waking consciousness.

Again, excursions from the critical regime are best seen in the exaggerated states of affective arousal and activation. In clinical elation (mania), cognition becomes expansive and grandiose (subjectively confident) to a fault. This reflects exaggerated, indeed pathological, integration. In clinical anxiety, cognition becomes highly restricted in scope to the ruminative, typically aversive, focus, as if consciousness is dominated by an extension of the inhibitory specification seen in NREM sleep, creating a pathological and unbalanced differentiation.

At criticality, conceptual organization appears to benefit from a balance of integrating and differentiating influences, allowing not only differentiation and articulation of elements and objects, but the hierarchic integration that allows abstract concepts [110,115]. In the statistical physics literature on criticality in complex systems, there may be a similar phenomenon in which attractor dynamics (which can be related to the organization of complex connectivity of transient cell assemblies) allow *multiscale* patterns [116]. The network of relations allows not only short-distance relations or long-distance relations, but dynamics that include multiple scales of effective connections within the network simultaneously. Furthermore, the network becomes *metastable*, so that there are not large variations in scale from moment to moment, but a maintenance of the multiscale patterns over extended intervals [116]. To the extent that consciousness reflects the complex dynamics of criticality, the structure of concepts may allow the metastable, multiscale network patterns that support both integration and differentiation of information, and thus abstract concepts, simultaneously.

## 10. The Background Limbic Variational Dynamics That Generate Conscious Experience

The evidence on the consolidation of memory in sleep provides fundamental insights into the neurophysiology of ongoing and complementary psychological self-organization in both sleep and waking. The dual NREM and REM memory systems provide unique and opponent forms of adaptive differentiation and integration of experience, respectively [33]. Because biological control has evolved with each learning and memory system, these might best be described as motive-memory systems. The Bayesian mechanisms of active inference precisely characterize this process, in which the organism’s daily and nightly self-organization is grounded in the excitatory consolidation of the Bayesian, ontogenetic priors of the self-world model in REM, in response to the automatic incorporation of the unpredicted constraints from the environment in the inhibitory mechanisms of NREM.

To this point we have described the major excursions of inhibitory differentiation of memory in NREM, complementing the excitatory integration of REM, and we hypothesized that it is the simultaneous operations of these motive-memory systems that allows the criticality of consciousness. Both memory systems are active in the background of waking consciousness. Their excursions in periods of rest, reflection, or mind-wandering may determine the unconscious background (the fringe of the **E**–**I** criticality of consciousness) that continues those neurophysiological mechanisms of memory organization that are exercised in their canonical forms in sleep.

We interpret these mechanisms of activity-dependent synaptic regulation of cognition to reflect the continuing self-organization of neural development [42]. In light of this developmental perspective, it may be appropriate to use Jean Piaget’s developmental model of cognition [117,118] to illustrate how ongoing unconscious conceptual operations in waking may continue the preparation for consciousness that is begun in each night’s inhibitory NREM and excitatory REM states.

Piaget described the child’s cognition as varying between *assimilation*, when experiences can be integrated within the child’s existing conceptual schemas (we might say Bayesian priors of predictive control), and *accommodation*, when reality does not fit the familiar expectations of the self, and concepts (self-evidencing) must be error-corrected by the new evidence. This model clearly frames conceptual self-organization in a predictive-corrective process that aligns well with active inference. This alignment can be extended to include not only the nightly reorganization of the self in NREM and REM memory consolidation, but also the waking processes of integrating ongoing active and reflective experience with variational excursions that bring the spontaneous and unconscious operations of the dual limbic memory systems—once they are adequately developed—into consciousness.

The neurophysiological mechanisms of these corticolimbic dynamics, including their extensive reliance on subcortical control, is gradually becoming clear. We can now align the oscillatory dynamics of corticolimbic networks with excitatory operations linked with primary dorsal limbic excitatory control (phasic arousal), including theta modulation of local gamma oscillations [119]. We can also characterize the mechanisms of inhibitory control, under primary ventral limbic and striatal-thalamic control, including beta and alpha oscillations [120,121,122]. With the recognition that local cell assemblies of columnar networks—and thus the attractor dynamics of cognitive and memory networks—are synchronized by both excitatory and inhibitory operations, the implication is that the ongoing cortico-limbic-striatal dynamics are cycling at these control frequencies (7 Hz, 10 Hz, 20 Hz), thereby constructing candidate network cell assembly attractors hundreds of times per minute.

What appears in consciousness may then be the unique stabilization and critical slowing of these unconscious corticolimbic dynamics of memory function during the ephemeral periods of **E**–**I** criticality. The contributions of active inference to ongoing cognition can then be understood as the variations between **E** and **I** control with differing contributions of the dual-motive-memory systems to the dominant, emergent predictive impulses and corrective constraints that are stabilized in those moments of criticality that yield distinct consciousness.

### 10.1. Excitatory Generation of Predictions

The generative process of organizing predictions may draw preferentially upon the contributions of the dorsal limbic division and its excitatory REM memory system in waking cognition [31]. Hobson emphasized a similar view as he described the REM-like protoconsciousness in creative thought [122] and in the generative process that constructs the predictive priors of active inference [114]. With the hippocampus at its base, the dorsal limbic division may be well suited to rehearse memory representations and fragments with hedonic significance for personal affordances [123,124], engaging the anterior cingulate loop to recruit pattern-completion in the CA3 region of the hippocampus, such as through the excitatory theta-nested gamma bursts [120] to organize a nascent prediction stream, pre-activating likely cortical states and extending the fringes of the specious present so that consciousness arrives to a field already rich with integrative gist.

In the framework of Hobson and Friston [114], these dorsal limbic operations would engage the REM-like memory system for the *primary cognition* that allows both fantasies and personally meaningful predictive schemas. Based on this REM-like feedforward memory system, operating from the holistic priors of personal affordances [30], this component of background memory is similar to Piaget’s *assimilation*. The unconscious activities of waking memory can be expected to proceed in ways that are congruent with the prior expectations of the self, recently organized in the nightly excitatory reorganizations of REM sleep.

### 10.2. Selective Inhibitory Specification

In a similar fashion, the cognitive process supporting waking active inference may be supported by ongoing dynamics of unconscious working memory that activate information relevant to constraint and error-correction through the inhibitory neurophysiology that has consolidated information uniquely in NREM sleep [33]. Particularly important to background ruminations may be the ventral limbic controls over the patch division of the striatum that uniquely regulates the midbrain inhibitory control over corticostriatal loops.

The ventral limbic cortices, amygdala, and paralimbic hippocampal fields converge on the patch (striosome) compartment of the ventral striatum, whereas multimodal isocortical and sensorimotor streams preferentially target the surrounding matrix [125]. Viral-tracing and connectomics now show that striosomes send dense, GABA-ergic projections to the dopamine (DA) neurons of the substantia nigra pars compacta (SNc) and ventral tegmental area (VTA), enabling the patch system to pause—and then rebound—midbrain DA output. In parallel, the medium-spiny neurons of the striatal matrix discharge along classic direct/indirect pathways toward pallidum and thalamo-cortical relays. Consistent with the recognition of ventral limbic self-regulation through collothalamic interactions with the midbrain [30], the midline collothalamic nuclei (paraventricular, reuniens, intralaminar) provide a complementary limbic input to both striosome (patch) and matrix, but they bias toward striosomes, giving the patch compartment a privileged window onto arousal/uncertainty signals that originate in the midbrain.

These mechanisms may be integral to the unconscious mechanisms of rumination and inhibitory specification in working memory that allow both the differentiation and causal attribution of unpredicted events. Because striosomes can veto SNc/VTA firing, they implement a high-level inhibitory gain control over dopaminergic teaching or enabling signals [125]. When cortical-limbic prediction errors accumulate, patch inhibition transiently suppresses tonic DA, sharpening the contrast for subsequent phasic bursts that pull the matrix—and its cortical partners—into a new synaptic configuration. This two-step process can be described as *inhibitory specification*: (i) a brief dampening of outdated information-action patterns, (ii) a rebound DA surge that stamps in a re-weighted action-outcome map. In network terms, patch activity first widens the landscape of accessible attractors, while matrix plasticity then instantiates the selected attractor once the critical balance is re-established.

Recognizing that the ventral limbic regulation of these striatal-midbrain inhibitory specification dynamics is integral not only to limbic but neocortical function, the inhibitory reset of feedback information patterns may be candidate mechanisms for information differentiation, object delineation, and Piagetian accommodation. Piaget’s accommodation process is the necessary updating of a child’s cognitive schema when reality refuses to fit. The patch–matrix mechanism of the striatum offers a neural mechanism. The patch/striosome mechanism silences midbrain DA, which marks the existing assimilation system insufficient to fit perception, thereby loosening synaptic priors and preparing the ground for change. Then, once an alternative expectancy proves predictive, matrix-driven corticostriatal loops engrain the revised sequence of states and actions. This mechanism, cycling with corticostriatal loops at the beta frequency, provides the background processing that allows anxious ruminations to gain purchase in the unconscious precursors of conscious reasoning.

### 10.3. The Variational Backstage for the Momentary Appearances of Conscious Criticality

The consolidation of memory in disparate stages of sleep allows the inhibitory (largely striatal-thalamic GABAergic) mechanisms of delineating and articulating unpredicted events to alternate with the excitatory (largely lemnothalamic and forebrain) mechanisms of re–integrating the predictive capacity of the organism [33]. In waking, these memory processes are more or less simultaneous across the full vertical hierarchy of the neuraxis (Figure 1 and Figure 7), and their continuing corticolimbic dynamics can be expected to provide the access to working memory that allows both spontaneous and deliberate forms of cognition and the attendant experiences of consciousness. During quiet rest, the dorsal limbic impulse drives associative breadth, and semantic distance increases, whereas inhibitory specification periodically tightens the evidence constraint, forcing conceptual differentiation.

The alternation yields a rolling dialectic in the variational corticolimbic dynamics of background preparations for consciousness. The impulsive operations of the REM memory system generate a predictive sweep across latent mental associations, leading to expansive and often hedonic daydreams, and occasional intuitive leaps in problem-solving. The constrained and focusing operations of the NREM memory system lead to reactive cuts of the associational matrix, sharpening on a critical feature and allowing this feature to be reconstituted through the patch-DA-matrix mechanism in a new causal context. This cycle, repeated hundreds of times per minute in the cortico-subcortical loops, beneath awareness, maintains criticality: enough redundancy for flexible meaning, enough selectivity for actionable clarity.

The result is the unconscious operations of working memory that process ongoing experiences to be enjoined in the conscious *perchings* of criticality. In this way, conscious experience can be seen as the momentary after-image of the unconscious processes of assimilation and accommodation.

## 11. Limitations and Directions for Future Research

While the criticality framework presented here offers a powerful formulation of consciousness as arising from the dynamic balance of integration and differentiation, it remains incomplete in its current form. Specifically, it lacks a mechanistic account of self-awareness—the subjectivity that defines conscious experience as being for someone. This limitation is not unique to the present model. Formal implementations of active inference have similarly been criticized for failing to explain the feeling of being—the intrinsic directedness and agency of phenomenal consciousness.

### 11.1. Affective Dynamics as the Structure of Subjectivity

We propose that this gap may be narrowed by introducing the role of affectively regulated excitation and inhibition as a computational substrate of intentionality. The propagation of excitation (**E**), particularly through dorsal limbic circuits, enables expansive, feedforward integration across the cortical hierarchy. This excitatory mode underlies elation, openness, and the anticipatory drive associated with intentional action. In contrast, inhibitory regulation (**I**), routed through ventral limbic and basal forebrain systems, imposes feedback constraint—shaping prediction by enforcing precision. Subjectively, this mode is associated with anxiety, focused vigilance, and the narrowing of experiential scope.

These affective states—elation and anxiety—are not secondary to cognition; they are the computational control systems that determine the scope and granularity of inference. They shape the balance of integration and differentiation, and in doing so, structure the architecture of experience. The self is not a separate processor; it is the coherently regulated field of these dynamics, evolving in real time.

### 11.2. Intentionality Disrupted: Absence Seizures and the Fragility of Self

Evidence from clinical neurophysiology offers a compelling demonstration of how these dynamics can fail. Absence seizures, characterized by transient lapses in behavior without postural collapse or overt loss of consciousness, reveal a specific disruption of intentionality. The person ceases speaking, moving, or engaging for many seconds, yet appears awake, upright, and unaware of their disconnection.

Electrophysiologically, these seizures involve spike-wave discharges across anterior thalamocortical components of the Papez circuit, disrupting feedforward processing in dorsal limbic networks. Unlike limbic seizures involving the hippocampus or amygdala, absence seizures preserve arousal but collapse the generative model of action. In essence, the self ceases to intend, though the body remains conscious.

This suggests that intentionality—and by extension, self-awareness—requires not only inferential updating, but a coherent excitatory drive from dorsal limbic systems, regulated by affect and situated within a hierarchical network architecture.

### 11.3. Multiscale Modularity as the Substrate of Self-Referentiality

As one of the reviewers of this paper rightly noted, **E**/**I** dynamics alone are insufficient to explain the recursive embodiment necessary for consciousness. What is required is a multiscale, modular architecture capable of supporting reentrant inference loops across temporal and spatial hierarchies. The brain meets this requirement.

The brain’s functional networks can be shown to be hierarchically modular, with stable substructures at multiple nested scales [126]. Alemdar and associates emphasize multiscale intermittency—transient, cross-scale instabilities that dynamically reconfigure global dynamics. These instabilities are not noise; they are likely the control surfaces by which high-level affect modulates lower-level computation [127]. Sbitnev [128] argues that self-referential intentionality emerges only when functional gradients support causally closed, scale–integrating loops—conditions satisfied only in complex, biologically embodied architectures.

In a multiscale framework as described by these authors, we would argue that self-awareness is not a symbolic operation, but a multiscale dynamical resonance, recursively sustained through affectively gated excitation and inhibition across hierarchical modular networks. The implicit, felt subject of experience is the locally coherent, globally embedded field of inference.

### 11.4. Future Directions: Toward Affective-Critical Neurocomputational Models of Selfhood

To simulate this structure computationally, models must go beyond symbolic active inference and incorporate multiscale nested networks with recurrent, reentrant dynamics. It will also be important to formulate affectively tunable **E**/**I** parameters that regulate integration and differentiation across levels. Finally, mechanisms for state persistence, attractor layering, and flexible modular switching will be necessary to implement hierarchical modularity in an effective neurocomputational framework.

Ultimately, we believe that the balance of elation and anxiety, implemented as dynamically regulated **E**/**I** control across hierarchical modules, constitutes a testable and generative model of the self. It provides a biologically grounded mechanism for intentionality, and a path forward for Bayesian neurocomputational modeling of consciousness.

## 12. Conclusions: Adaptive Control of Variational Criticality

In our survey of the neurophysiological mechanisms of consciousness, we propose that the memory systems for supporting working memory in waking cognition are grounded in the excitatory and inhibitory corticolimbic dynamics whose mechanisms are revealed in the alternations between NREM and REM sleep. As they are engaged in waking cognition, these memory systems bring their inherent motive biases, the integral motive biases of the dorsal and ventral limbic systems [31,33], to regulate the criticality of consciousness.

In waking experience and behavior, the dual memory systems operate simultaneously in what we propose is a close balance that achieves the efficient control stability and information processing capacity of **E**/**I** balance. This balance appears most flexible when it includes adaptive excursions, such that a dominance of **E** allows the dorsal limbic system to regulate the entire synaptic network with a feedforward bias toward generative predictions, and at other times, the dominance of **I** allows the network to be regulated by the inhibitory control of the ventral limbic division. It is this adaptive control, exerted largely by the ongoing and largely unconscious operations of the dual memory systems, that we describe as the criticality that generates consciousness.

Thus adaptive, motivated control is integral to the operation of each memory system. In NREM sleep, the replay of significant events, delineated through spindle-coupling of hippocampal sharp waves, and overall inhibitory control through slow oscillations, are the candidate mechanisms for engaging the explicit memory consolidated in NREM. These are mechanisms for organizing the synaptic cell assemblies that constitute memories, through the ventral limbic inhibitory forms of control, associated with the motive bias of anxiety. The fact that unpredicted events gain some unique identification—that flags them for the specific NREM delineation and consolidation in sleep—suggests that some kind of limbic resonance during waking prepares the representation of these events for priority NREM processing [33]. It is consistent with this mechanism to propose that the waking process of explicit recall during ongoing cognition is also engaged by similar mechanisms of limbic inhibitory control, differentiated, articulated, and stabilized in the informatic status quo by the affective control of anxiety (**I**). NREM sleep thus prepares the waking unconscious mind for the continuing support it provides to waking working memory and the preconscious preparations for conscious thought.

In a different way, the predictive control bias of the dorsal limbic division appears to be integral to the excitatory consolidation of REM sleep. Although content reports of REM dreams include many negative and threatening events [129], certain findings suggest that the reorganization or reconsolidation of existing memories in REM sleep includes a positive adaptive bias (**E**) that endows the reorganized priors to engender predictions aligned with the organism’s adaptive coping efforts. Thus after aversive stimulation in rodent studies, followed by re-exposure to the same context without aversive stimulation, REM appears essential to what is called *extinction memory*, the formation of an overlay or adaptation of the older aversive memory for the new, more recent non-aversive memory of the context [130]. Importantly, the original aversive memory is not lost, as evidenced by its rapid restoration by new training. Observing the disruption of REM in human post-traumatic stress disorder (PTSD), Pace-Schott and associates have suggested that effective sleep is protective for PTSD through REM’s capacity to reframe (repress) aversive experiences through a process analogous to extinction memory. This positive adaptive bias may be integral not only to the memory reconsolidation of REM, but to the dorsal limbic memory system’s contribution to the feedforward (forward-looking) control of waking cognition.

### 12.1. Experimental Predictions

Perhaps the clearest experimental prediction is that there are dual memory systems, with integrating versus differentiating capacities, and they are inherently motivated by elation (**E**) and anxiety (**I**), respectively. There are increasingly robust measures of the neurophysiological oscillations of cortical excitation and inhibition for testing the hypothesis of criticality, such as with measures of theta-gamma coupling for excitation and alpha-beta modulation for inhibition [120,121].

With behavioral measures, the current cognitive neuroscience literature includes increasingly sophisticated paradigms for examining neural mechanisms of memory, such as are contrasted by broad generalization versus remembering specific features [131]. With appropriate experimental and neurophysiological measures, the prediction can be tested that the motive bias of **E** (elation) is integral to the feedforward REM memory (generalization) whereas the control system of **I** (anxiety) is essential for the feedback NREM (specification) memory system.

### 12.2. Phenomenological Implications

Subjectively, because the dual memory systems are balanced at criticality, consciousness may appear to maintain affective neutrality. However, for criticality, adaptive shifts must occur in the unconscious background mechanisms of memory, according to our formulation, which may be sensed by the furtherances and hindrances, as subtle changes in the affective quality of subjective experience. For example, prior to a creative insight, a brief shift toward **E** and integrative thinking may allow the expansive (*protoconscious*) apprehension of novel associations [122]. When it is drawn into criticality and stabilized by balanced inhibitory control, this novel construction would be experienced as a sudden insight, with the attendant effect of some degree of elation. Importantly, a shift toward **I** dominance (with the attendant quality of anxiety) may indeed be necessary in order to stabilize this new insight and allow it to be sufficiently focused for retention (including in subsequent NREM sleep). As a result of these preparatory excursions in the process of working memory—involving both optimistic creative prediction and critical focused correction—we may find the most objective reasoning to require variations from criticality to achieve these forms of affective self-regulation.

Through recognizing the nature of the dual memory systems, the hypothesis of consciousness as *integrated* information [3] can indeed be tested, both experimentally and experientially. Thus, according to our analysis, information integration in consciousness requires the excitatory control of the REM memory system. Interestingly, however, the feedforward REM-consolidated memory system appears *not* to contribute to the explicit memory associated with focal, declarative consciousness of qualia. Rather, excitatory REM consolidation in sleep (and we would expect the continuation in waking) supports the *implicit* memory that is non-declarative [87]. If consciousness is defined as explicit qualia, as often seems the case, then integrated information theory would not only be falsifiable, but falsified.

Instead, to appreciate the more implicit experience that facilitates information integration may require a broader appreciation of the intuitive and non-declarative, preverbal states of consciousness [47,50,132] where integrated information seems to be achieved as an essential capacity of experience.

Conversely, if consciousness is associated with *differentiated* information [3], then the explicit declarative qualia can indeed be aligned with the differentiating memory system, which can now be identified with the inhibitory neurophysiology of NREM sleep.

### 12.3. The Entropic Nadir of Criticality

Because the dual memory systems are organized through their motivationally biased excitatory and inhibitory neurophysiological mechanisms, both subjective and objective qualities of consciousness may be explained by the variations in these mechanisms, differentiating experience at times through inhibitory specification and constraint, and integrating it at other times through the holistic scope of excitatory control. Yet as critical as these variations are for adaptive control, enacting the affective control of experience through each day’s conceptual variations and each night’s **E**/**I** excursions in memory organization, they reflect both energetic effort and subjective need bias in proportion to their deviation from balance. Only at the exact balance at **E**/**I** criticality is free energy minimized sufficiently [23] to allow the control sensitivity for optimal subjective consciousness to emerge.

## Figures and Tables

**Table 1:** Key Terms.

Term/Symbol	Concise Definition	Typical Biological/Computational Correlate
Feed-forward (limbifugal, generative) projections	Propagation of descending predictions from higher to lower cortical levels; sets up hypotheses about upcoming input.	Cortico-cortical projections from infragranular to supragranular layers; hippocampal → neocortical replay; θ–γ “driver” coupling.
Feedback (limbipetal, corrective) projections	Error-correcting signals that compare predictions with sensory input and update beliefs via ascending prediction errors.	Cortico-cortical projections layer 2–3 → 4; thalamo-cortical bursting; α–β “modulator” rhythms.
Excitatory/Inhibitory (**E**–**I**) balance	Dynamic trade-off between excitatory drive and inhibitory restraint that keeps networks near criticality.	Pyramidal–interneuron loops; spike-time cross-correlations; LC-NE vs. VTA-DA neuromodulation.
Criticality	An operating regime poised at the transition between order and disorder, maximizing dynamic range and information flow.	Power-law avalanche size distributions in hdEEG/MEG; 1/f spectral slope.
Markov blanket	The statistical boundary or interface that shields internal states from external states while permitting exchange.	Boundaries between each hierarchical level of the Structural Model; thalamo-cortical loops.
Variational Free Energy	Upper bound on surprise (i.e., self information); minimized when the agent’s generative model matches sensory data.	Evidence-lower-bound (ELBO) in Bayesian machine-learning; precision-weighted prediction error in predictive coding and Bayesian filtering.
NREM (N1–N3)/REM	Alternating sleep macro-states: NREM supports synaptic down-selection and memory replay; REM integrates and contextualizes mnemonic traces.	Slow oscillations/spindles (NREM) vs. θ-γ coupling and PGO-waves (REM).

**Table 2:** Definition of symbols used in the formulation of the **E**–**I** gain control.

*Symbol*	*Definition*	*Interpretation*	*Possible Neural Mechanism*
o	Sensory observations	Data to be explained	Primary sensory afferents
s	Hidden (latent) causes/states	The causes of sensory input	States of affairs in the world
q(s)	Variational posterior	Posterior belief distribution in response to sensory evidence	Synaptic activity in cortical hierarchies and thalamocortical loops
p(s)	Prior over s	Prior beliefs or constraints	Synaptic activity and efficacy
**E**	Excitatory gain (i.e., prior precision)	Dorsal limbic, lemnothalamic regulation	Pontine θ–γ multiplexing, ACh, phasic LC-NE
**I**	Inhibitory gain (i.e., sensory precision)	Ventral limbic, collothalamic regulation	thalamic/striatal α/β bursts, tonic LC-NE, VTA D2/D4

**Table 3:** Regulation of Precision in Active Inference.

Gain	Precision	Computational Effect		Oscillatory/Neurochemical Correlate
E ↑	Prior precision	Explores narrower range of priors (reduced conceptual scope with increased central coherence)		θ–γ multiplexing, ACh, phasic DA & LC-NE
I ↑	Sensory precision	Precise, longer-lived responses to sensory perturbations		α/β bursts, tonic LC-NE, D2/D4
ρ = **E**/**I**	Precision ratio	Self-reliance vs environment reliance		Day-dreaming vs reality-checking
Criticality	Regime in which **E** and **I** are balanced, so that ρ hovers near the critical regime across the diurnal cycle.		**E**–**I** phase portrait spiraling onto a limit-cycle at criticality.	

**Table 4:** Memory consolidation effects of NREM and REM sleep stages, complementing the neurophysiologic mechanisms of active inference in the nightly consolidation of memory.

Phase	Precision	Network Consequence	Behavioral Outcome
Early NREM	**I** dominates	Sharpens synaptic weights; down-selects noisy traces	Declarative memory stabilization
REM burst	**E** dominates	Integrates remote associations	Insight, emotional tagging
Late-night REM dominance	ρ oscillates near optimum **E**/**I** balance	Network at ρ ≈ 1	Next-day critical readiness

**Table 5:** Neurophysiological mechanisms of NREM and REM sleep in regulating cognition and experience.

Stage	Prior Precision E	Sensory Precision I	Computational Work	Phenomenological Residue
Stage-2 NREM (spindles)	↘ moderate	↗ moderate	Event segmentation: spindles carve the day’s stream into ~0.5 s packets. Hippocampal ripples that co-occur under a spindle trough bind elements that consistently follow one another—extracting hidden probabilistic contingencies.	Morning “aha” moments about order (“the light blew because the breaker was already flipped”).
Slow-wave NREM (SWS)	minimal	maximal	Hierarchical nesting: each slow-wave UP state embeds multiple spindles which embed ripples, building multi-timescale *directed graphs* of causality in medial prefrontal cortex.	Feeling of settled chronology; fewer temporal confusions in recall.
Phasic REM	↑↑ broad	↓↓ brief	Generative replay: pontine P-waves switch cortex into model-sampling. Weakly linked spindle chunks co-activate, testing counterfactual chains. Dopamine tags successful free-energy-reducing linkages.	Vivid dream scenes where remote ideas collide; creative insights on waking.
Tonic REM tail	moderate	moderate	Re-binding: LC/orexin micro-blips nudge sensory precision upward, copying REM-tested linkages back into hippocampus.	Dream coherence peaks just before awakening—memorable story line.

## Data Availability

No new data were created or analyzed in this study.
